# Cranial Morphology of the Carboniferous-Permian Tetrapod *Brachydectes newberryi* (Lepospondyli, Lysorophia): New Data from µCT

**DOI:** 10.1371/journal.pone.0161823

**Published:** 2016-08-26

**Authors:** Jason D. Pardo, Jason S. Anderson

**Affiliations:** Department of Comparative Biology and Experimental Medicine, University of Calgary, Calgary, Alberta, Canada; Institute of Vertebrate Paleontology and Paleoanthropology Chinese Academy of Sciences, CHINA

## Abstract

Lysorophians are a group of early tetrapods with extremely elongate trunks, reduced limbs, and highly reduced skulls. Since the first discovery of this group, general similarities in outward appearance between lysorophians and some modern lissamphibian orders (specifically Urodela and Gymnophiona) have been recognized, and sometimes been the basis for hypotheses of lissamphibian origins. We studied the morphology of the skull, with particular emphasis on the neurocranium, of a partial growth series of the lysorophian *Brachydectes newberryi* using x-ray micro-computed tomography (μCT). Our study reveals similarities between the braincase of *Brachydectes* and brachystelechid recumbirostrans, corroborating prior work suggesting a close relationship between these taxa. We also describe the morphology of the epipterygoid, stapes, and quadrate in this taxon for the first time. Contra the proposals of some workers, we find no evidence of expected lissamphibian synapomorphies in the skull morphology in *Brachydectes newberryi*, and instead recognize a number of derived amniote characteristics within the braincase and suspensorium. Morphology previously considered indicative of taxonomic diversity within Lysorophia may reflect ontogenetic rather than taxonomic variation. The highly divergent morphology of lysorophians represents a refinement of morphological and functional trends within recumbirostrans, and is analogous to morphology observed in many modern fossorial reptiles.

## Introduction

The Paleozoic origins of modern lissamphibians (Caudata, Anura, and Gymnophiona) have been a matter of substantial debate for over a hundred years. Recent effort has resulted in an emerging consensus that modern lissamphibians are amphibamid temnospondyls [1–6] on the basis of cranial anatomy, dental morphology [7], inner ear morphology [1], and life history [6,8–10]. However, the substantial morphological gap between amphibamids and the earliest representatives of the Caudata [11], Anura [12,13], and Gymnophiona [2,14] has left room for skepticism of this prevailing view. A second clade of Paleozoic tetrapods, the Lepospondyli, has been identified as a possible stem-group of all lissamphibians [15–17] or of the Gymnophiona specifically [3,18–21], on the basis of general similarity of the palate and postcranial skeleton, and extent of cranial ossification.

Within the Lepospondyli, specific attention has been given to the Lysorophia, a group of lepospondyls with elongate bodies and reduced limbs. Early reports of lysorophian fossils identified these animals as early aquatic salamanders [22–23] or caecilians [24] but the first major attempts at describing lysorophian anatomy made it clear that lysorophian anatomy is inconsistent with direct ancestry to any specific lissamphibian group, and that lysorophians are either relatives of Lissamphibia more generally [25] or are morphologically-specialized early tetrapods without clear affinities to any modern tetrapod group [26–28]. A close relationship between lysorophians and modern lissamphibians has reemerged in some phylogenetic analyses [15–16, 29] and individual characteristics of lysorophians, such as the zygokrotaphic skull, have reemerged as possible support for a close relationship between lysorophians and caecilians [19] (but see [30] for further discussion), but a majority of analyses continue to find lysorophians to have no direct relevance to questions of lissamphibian origins [1–5, 21, 31].

In addition to an uncertain relationship between lysorophians and lissamphibians, the relationships of lysorophians within Lepospondyli are also unclear. A number of workers have suggested a relationship between lysorophians and recumbirostran ‘microsaurs’ (a clade of lepospondyls that includes pantylids, gymnarthrids, ostodolepids, and brachystelechids [3]) on the basis of occipital, vertebral, and pectoral morphology [28, 32], but a close phylogenetic relationship between these taxa has only been recovered by a minority of analyses [15–17]. Instead, a majority of analyses place lysorophians within a larger assemblage of ‘lepospondyls’ with elongate bodies and reduced limbs, alongside the Nectridea and Aïstopoda [3–4, 21, 31, 33–34]. Neither of these results are particularly satisfying. Whereas a relationship between lysorophians and recumbirostrans makes intuitive sense on the basis of vertebral, occipital, and appendicular morphology, this relationship lacks wholesale support within recent lepospondyl phylogenies. Alternately, a relationship with aïstopods and nectrideans is generally supported by recent phylogenetic analyses, but these taxa show few consistent similarities beyond general body shape and vertebral morphology, and much of this topology appears to be driven by characters describing skeletal reduction and axial elongation.

The difficulties of resolving the relationships of lysorophians to Lissamphibia and to other lepospondyl taxa both stem from general reduction of the skeleton and loss of morphology characteristic of other major groups. Anderson [21] has noted that this problem may be intractable due to methodological biases in datasets overwhelmed by convergence in “loss” characters. Similar intractable phylogenetic problems have been solved in lungfishes [35–36], caecilians [2, 37–38], and crocodilians [39] by studying variation in the often poorly-described but character-rich braincase. Recent studies have focused attention on the braincase of some lepospondyls [33, 40–43] and have identified variation that may be indicative of relationships.

Despite the potential importance of lysorophians in early tetrapod phylogeny, intensive study of the braincase has been lacking. This is, in large part, due to the preservational quality of the majority of lysorophian fossils available for study. Most well-preserved lysorophian fossils come from either Upper Carboniferous cannel coals, such as the Upper Freeport Coal (Allegheny Group, Upper Carboniferous) of the Dunkard Basin, or from the Lower Permian redbeds of Oklahoma and Texas. The two-dimensional preservation of the cannel coal specimens, and the uniform radio-opacity of the redbeds specimens, makes further study of this material using new imaging techniques (such as μCT) difficult.

Abundant new lysorophian material has recently been reported from terrestrial paleosol horizons within the earliest Permian Eskridge Shale of Nebraska [44] and Speiser Shale of Kansas [45–46] within the Council Grove Group (**CG**G), representing lowstand sequences within a larger sequence of alternating terrigenous and nearshore marine sediments. Vertebrate bone from these deposits typically shows little diagenetic alteration, and the surrounding matrix generally exhibits little to no diagenetic precipitation of iron (unlike redbeds fossils), a fact which has made possible detailed study of **CG**G fossils using micro-computed x-ray tomography (μCT) [33, 36]. Lysorophians are the most common tetrapods in vertebrate-bearing horizons in **CG**G paleosols and are generally found partially or completely articulated, typically within flask-shaped burrow structures [44–46]. Many well-preserved, three-dimensional skulls have been collected from these localities, permitting detailed study of braincase morphology in *B*. *newberryi* at a range of sizes. Here we describe in detail the morphology of the skull and braincase of a partial ontogenetic sequence of these skulls from the Council Grove Group using μCT.

## Materials and Methods

### Geological Context

The **CG**G spans the latest Carboniferous (Gzhelian) through the earliest Permian, representing the entirety of the Asselian in Kansas and Nebraska, and possibly extending into the Sakmarian [47–48]. These sediments record a series of fifth-order transgression-regression sequences bounded by well-developed fossil soils representing low-stand deposits. These fossil soils, which are typically gray-green, but sometimes also reddish-brown or mottled, are classifiable as vertisols or aridisols [49] and represent periodically waterlogged muds with limited organic content and high groundwater influence. Traces of roots [49] and vertebrate burrows [36, 44–46, 50] have been reported from these fossil soil horizons. Fossil vertebrates are abundant in these horizons, including gnathorhizid lungfishes [36, 44, 50], dvinosaurian temnospondyls [44, 51–54], amphibamid temnospondyls [44, 55], recumbirostran ‘microsaurs’ [33, 44, 56], diplocaulid nectrideans [57, 58], and rare diadectids and synapsids [44, 57], as well as numerous specimens of the lysorophian *Brachydectes newberryi*. These vertebrate-bearing localities have been interpreted as seasonal wetland systems [44, 46], similar to either vernal pools or playa lakes. The vertebrate assemblage at these localities appears to be largely autochthonous, although some taxa (such as the synapsids, diadectids, and recumbirostran ‘microsaurs’) may have been only occasional users of the wetland system and are represented primarily by rare isolated fragments (but not always, see [33]), in comparison with abundant obligately aquatic species (such as lungfishes), which are typically found articulated in burrow structures likely associated with aestivation [36, 44, 50, 59]. Lysorophian fossils from the **CG**G are typically found articulated in burrow structures as well [44–46] but generally occur at the margins of inferred ephemeral pond deposits [46] along with other tetrapods [59], whereas burrows of gnathorhizid lungfishes are typically concentrated at the center of these ponds [59].

**Fig 1:**
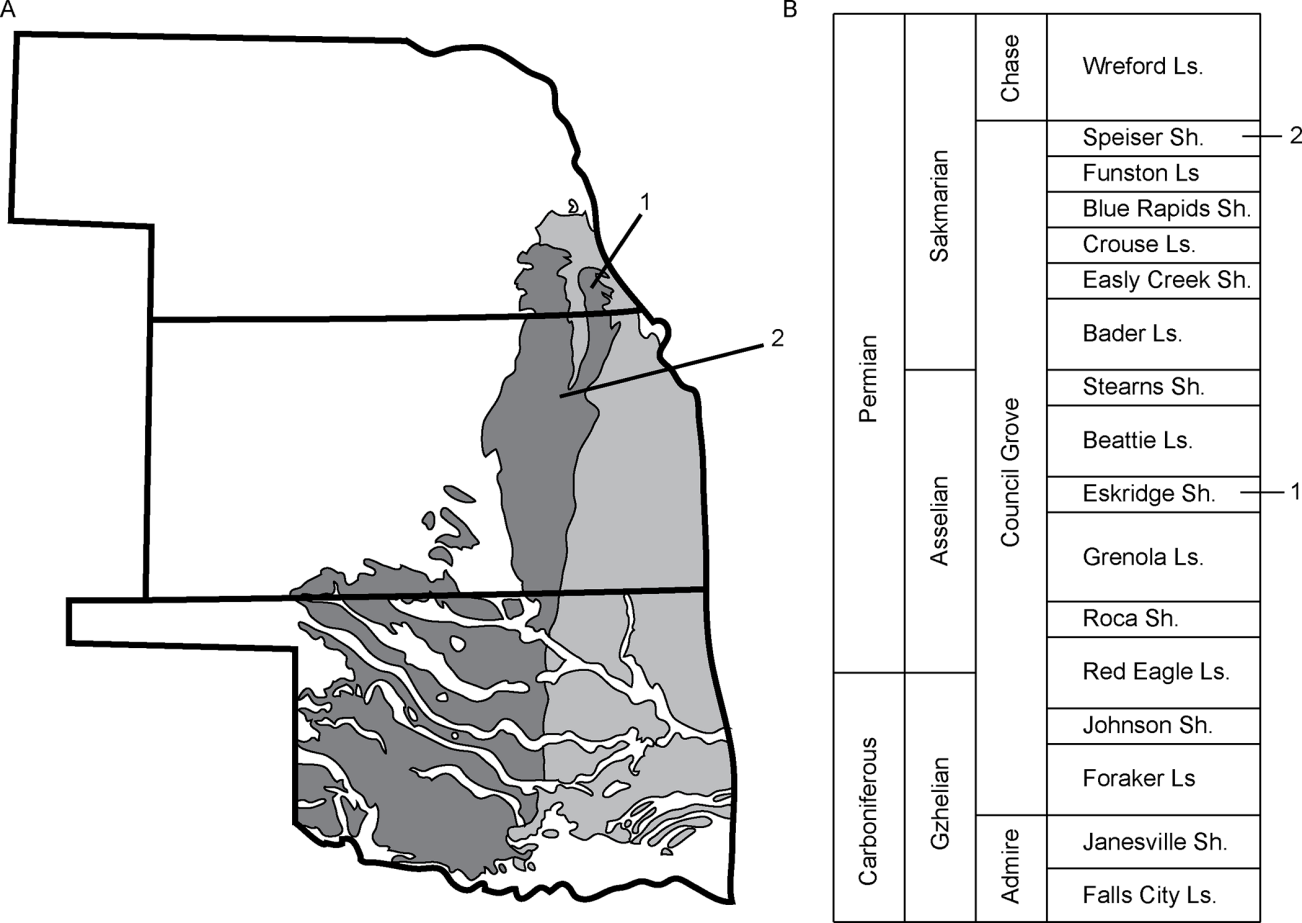
Stratigraphic and geographic context of specimens studied. **A**, geographic location of specimen localities; **B**, stratigraphic position of localities, after [48]. **1**, Humboldt, NE, localities (undivided); **2**, Eskridge, KS locality. Light gray indicates Upper Carboniferous surface deposits; dark gray indicates Lower Permian surface deposits.

Fossils of *Brachydectes* sampled in this study were collected from two vertebrate-bearing horizons in Lower Permian deposits of the upper **CG**G, one within the Eskridge Shale, and one within the Speiser Shale (Fig 1). Fossils from the Eskridge Shale come from a single horizon (Paleosol 2 of [49]), which has been sampled at three fossil-bearing localities in Richardson County, Nebraska, collectively the Humboldt Localities [44]. The Eskridge Shale is well-constrained to the early Asselian on the basis of marine microfossil biostratigraphy [47–48]. Fossils from the Speiser Shale come from a single horizon at a single locality west of Eskridge, Kansas. The Speiser Shale is constrained to the early Sakmarian [48] on the basis of conodont biostratigraphy. No permits were required for the described study, which complied with all relevant regulations.

### Specimens studied

**Fig 2:**
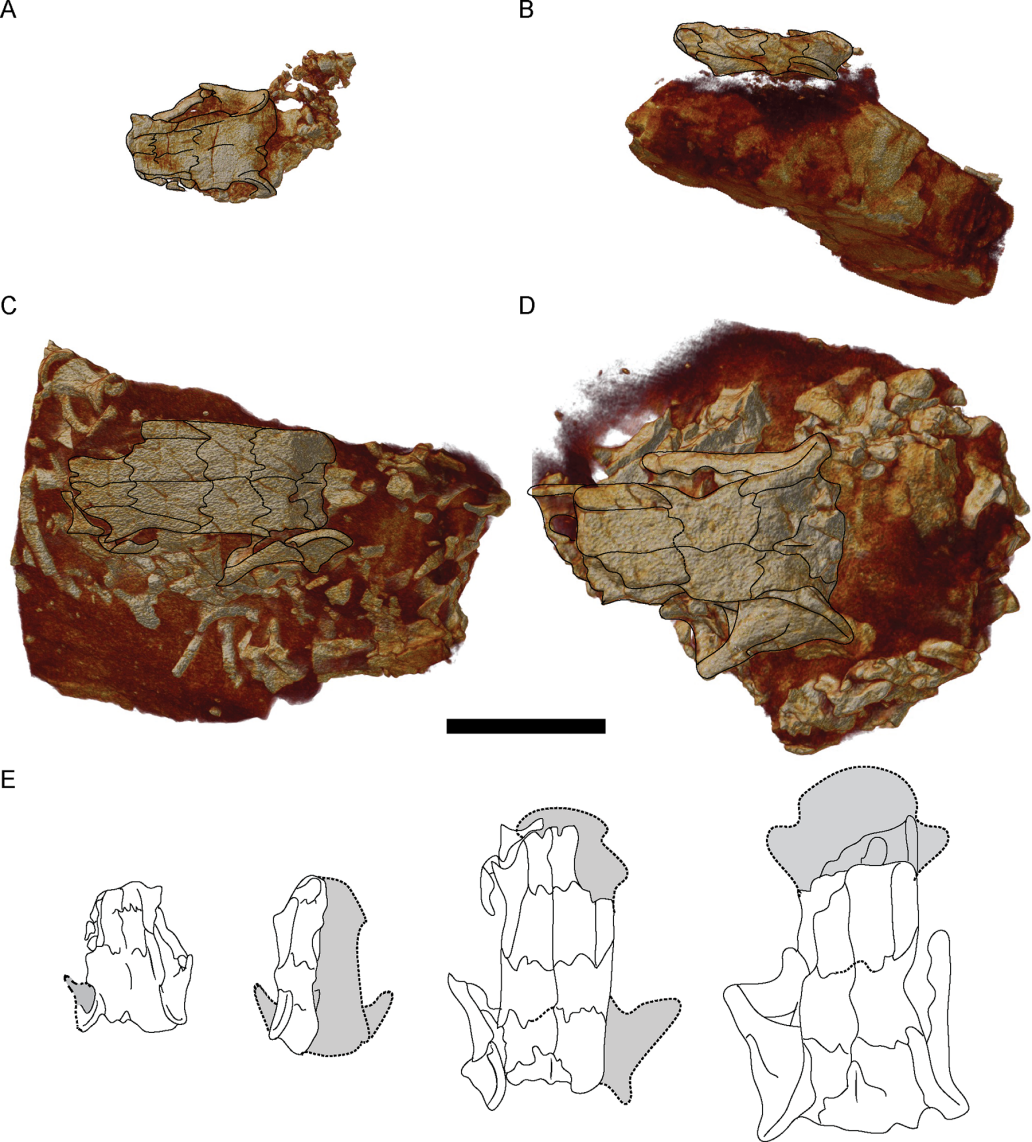
Volume renders of selected skulls studied, showing representative size variation in specimens, dorsal view. **A**, DMNH 47854; **B**, DMNH 521121; **C**, KUVP 49541; **D**, UNSM 32149; **E**, comparison of skull sizes. Scale bar equals 1 cm.

To fully image internal morphology of the skull of *Brachydectes newberryi*, we scanned nine skulls attributable to this species (S1 Table), representing the full range of sizes observed in the Council Grove Group (Fig 2). These specimens represent the bulk of Council Grove Group lysorophians from the Denver Museum of Nature and Science, Denver, Colorado (DMNH), the University of Kansas Natural History Museum and Biodiversity Institute, Vertebrate Paleontology Division, Lawrence, Kansas (KUVP), and the University of Nebraska State Museum, Lincoln, Nebraska (UNSM). All specimens were scanned in a Skyscan1173 conical beam desktop μCT (Bruker Corporation) and reconstructed as a stack of transverse slices in NRecon v. 1.6.6.0 (Skyscan, 2011). The resulting image stacks were downsampled using an interval of two and cropped in ImageJ using the virtual stack function to reduce computation burden. We then imported the resulting image stacks into Amira 5.3.3 (Visage Imaging, Inc.) for visualization in 3D. Volume renders of complete specimens were produced using the Volren module. Individual elements were then segmented using the LabelField module. Segmented elements were then isolated using the Arithmetic module and rendered using the Volren module, or were rendered as a three-dimensional surface using the SurfaceGen module.

### Braincase anatomy

In our study of the braincase of *Brachydectes*, we deviate from the traditional practice of describing individual bones and instead describe braincase morphology in terms of embryonic cartilaginous precursors of neurocranial ossifications. This approach has been previously employed in the study of the recumbirostran braincase [43] as it offers several advantages over traditional descriptive approaches. First, general braincase morphology is broadly conserved among tetrapods [60] permitting broad comparability between taxa on the basis of topological relationships between conserved structures (pathways of cranial nerves, presence of fenestrae, etc.). Second, this approach avoids a priori assumptions of homology between ossifications of the braincase in similar locations. Third, this facilitates comparison of conserved structure that may be preserved within nonhomologous elements, allowing us to focus on morphology of the structure rather than nomenclature of the bone. Finally, some lepospondyl taxa (e.g. the brachystelechids *Carrolla craddocki* [41] and *Quasicaecilia texana* [43], and phlegethontiids, [40]) as well as some lissamphibians (e.g. Gymnophiona, [37, 38]) exhibit massively co-ossified otoccipital regions (an *os basale*), and many early tetrapods massively co-ossified their braincase more generally [61–63], making description of braincase structure rather than individual bones necessary for any comparative efforts which include these taxa.

We do, however, recognize individual bones where possible, following the revised terminology of recent studies of recumbirostran morphology [41–43]. This allows us to recognize common ossification centres as well as identify which ossification center has expanded into a particular cartilage. By reporting both the structure of the cartilaginous precursors and the ossifications they are incorporated into, we maximize the available anatomical data in order to best understand the anatomy and relationships of *Brachydectes*.

## Systematic Paleontology

Lepospondyli Zittel 1888

Recumbirostra Anderson 2007

**Molgophidae Cope 1875**

Cocytinidae Cope 1875

Paterosauridae Broili 1908

Lysorophidae Williston 1908

**Revised diagnosis.** Elongate recumbirostrans with the following characteristics: large temporal emargination present contacting the parietals, temporal emargination confluent with orbit due to loss of posterior circumorbital bones (postfrontal, postorbital, and jugal), squamosal narrow and anteriorly-canted, orbitosphenoids and pila antotica robust and oriented vertically, ectopterygoids absent, pineal foramen absent, quadratojugal absent, intercentra absent, cultriform process of parasphenoid broad and rectangular.

**Phylogenetic definition.** Tetrapods more closely related to *Brachydectes newberryi* than to *Carrolla craddocki*, *Rhynchonkos stovalli*, *Dvellecanus carrolli*, *Cardiocephalus sternbergi*, *Euryodus primus*, *Huskerpeton englehorni*, or *Pantylus cordatus*. This is a stem-based definition.

**Discussion.** There has been a great deal of uncertainty in the literature concerning the correct name for the family containing *Brachydectes*. A number of familial designations have been employed in the literature, including Molgophidae [64], Lysorophidae [23], Paterosauridae [65], and Cocytinidae [64]. Of these names, Molgophidae appears first in the literature [64]. In a revision of the taxon, Wellstead [28] elected to maintain the name Cocytinidae, but it is unclear why, because there is no greater history of use of the name Cocytinidae and no lapse in usage of the name Molgophidae would necessitate suppression of the family-group Molgophidae in favor of Cocytinidae [ICZN Article 23.9.1]. Although *Molgophis* is now considered a junior synonym of *Brachydectes*, Article 40.1 of the ICZN states that synonymy of the type genus is not alone reason to change the family-group name. We therefore recommend that subsequent works use the name Molgophidae and treat Cocytinidae, Lysorophidae, and Paterosauridae as junior synonyms.

We deviate from previous workers by not recognizing the Lysorophia as a distinct order. Although phylogenetic position of *Brachydectes* is under some debate, the possibility exists that *Brachydectes* falls well within ‘Microsauria’ [17] and possibly within Recumbirostra [43], and its divergent morphology may represent extreme specialization within Recumbirostra, rather than a distinct lineage separate from Recumbirostra.

***Brachydectes*** Cope 1868

*Molgophis* Cope 1868

*Cocytinus* Cope 1871

*Pleuroptyx* Cope 1875

*Lysorophus* Cope 1877

**Type Species.** *Brachydectes newberryi* Cope 1868

**Diagnosis.** As for Molgophidae.

***Brachydectes newberryi* Cope 1868**

*Molgophis macrurus* Cope 1868

*Cocytinus gyrinoides* Cope 1871

*Pleuroptyx clavatus* Cope 1875

*Molgophis brevicostatus* Cope 1875

*Lysorophus tricarinatus* Cope 1877 in part

*Brachydectes elongatus* Wellstead 1991 in part

**Holotype.** American Museum of Natural History (AMNH) 6941, partial skull.

**Holotype locality.** Cannel coal above the Upper Freeport Coal (Allegheny Group, Moscovian, Pennsylvanian) of Linton Diamond Mine, Jefferson County, Ohio [66, 67].

**Attributed specimens.** Mayer Quarry (Humbolt, Richardson County, Nebraska, USA), Eskridge Shale (Council Grove Group, Asselian): DMNH 36599, right dentary; DMNH 43125, right dentary; DMNH 43224, skull with complete axial skeleton; DMNH 47854, skull with partial vertebral column; DMNH 49902, partial skull and partial skeleton; DMNH 50142, partial skull; DMNH 51121, skull with partial skeleton; DMNH 51122, partial skull with disarticulated skeleton; DMNH 52081, partial skull and skeleton. Shot in the Dark Quarry (Humbolt, Richardson County, Nebraska, USA), Eskridge Shale (Council Grove Group, Asselian): UNSM 32100, partial skull; UNSM 32115, partial skull; UNSM 32147, partial skull with partial skeleton; UNSM 32149, partial skull and associated postcranial elements. Eskridge Locality (Eskridge, Waubunsee County, Kansas, USA), Speiser Shale (Council Grove Group, Sakmarian): KUVP 49537, skull and partial skeleton; KUVP 49538, skull and partial skeleton; KUVP 49539, skull and partial skeleton; KUVP 49540, skull and partial skeleton; KUVP 49541, skull and partial skeleton.

**Revised diagnosis.** As for family. We provisionally synonymize all molgophids here as *Brachydectes newberryi*, as existing diagnoses are insufficient to distinguish between identified species. The presence of populations with vastly different numbers of trunk vertebrae [28] strongly suggests the presence of multiple species of molgophid in the Carboniferous-Permian if North America, but how this character is distributed amongst name-bearing specimens is difficult to ascertain.

## Description

### Skull Roof and Cheek

**Fig 3:**
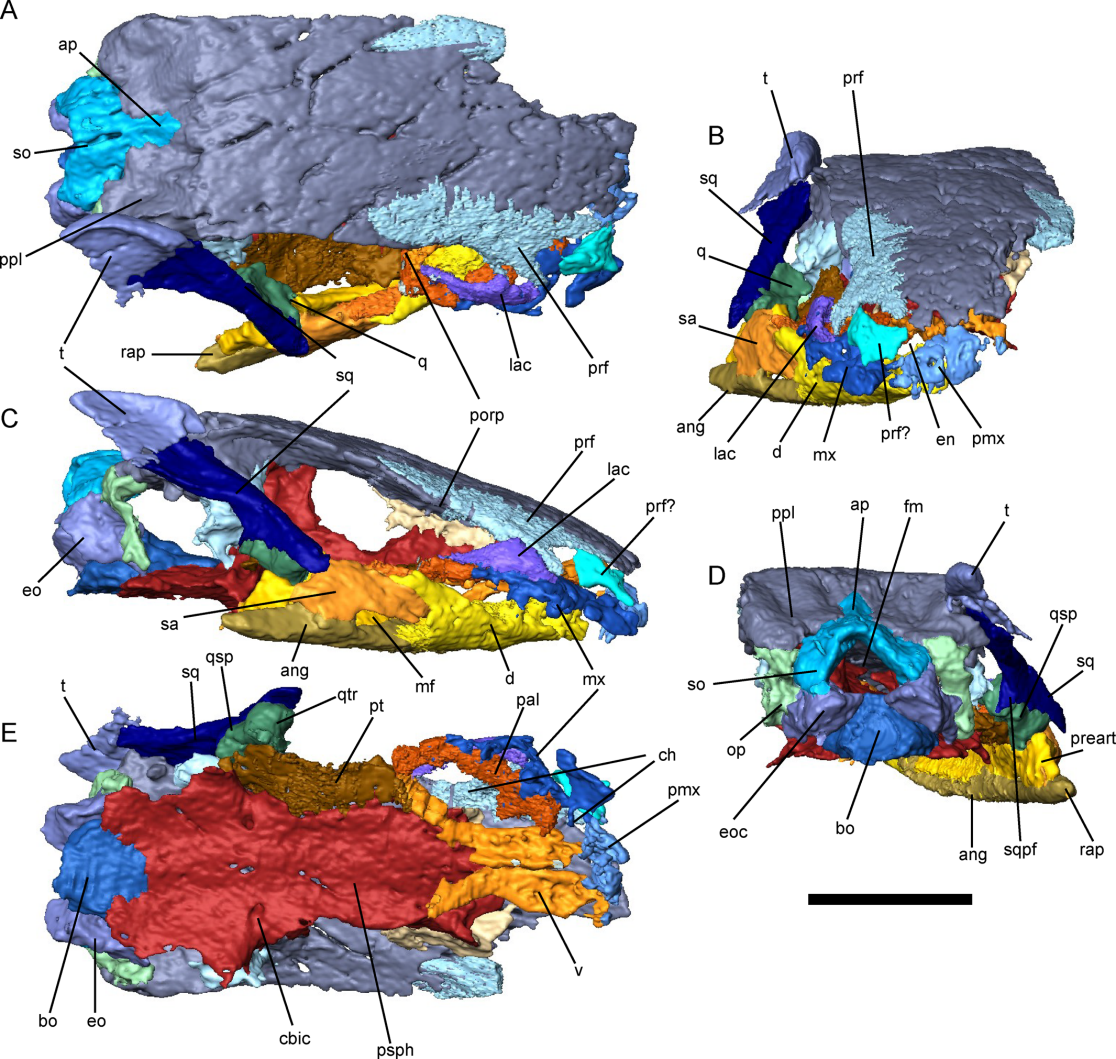
External skull morphology of *Brachydectes newberryi* KUVP 49541, rendered from μCT. **A**, dorsal view; **B**, anterior view; **C**, right lateral view; **D**, occipital view; **E**, palatal view, with lower jaws removed. Scale bar equals 5 mm. Abbreviations: **ang**, angular; **ap**, median ascending process of supraoccipital; **bo**, basioccipital; **cbic**, foramen serving the cerebral branch of the internal carotid artery; **ch**, choana; **d**, dentary; **en**, external naris; **eo**, exoccipital; **eoc**, exoccipital condyle; **fm**, foramen magnum; **lac**, lacrimal; **mf**, mandibular fenestra; **mx**, maxilla; **op**, opisthotic; **pal**, palatine; **pmx**, premaxilla; **porp**, postorbital process; **ppl**, postparietal lappet; **preart**, prearticular; **prf**, prefrontal; **psph**, parasphenoid; **pt**, pterygoid; **q**, quadrate; **qsp**, stapedial process of quadrate; **qtr**, trochlea of quadrate; **rap**, retroarticular process; **sa**, surangular; **so**, supraoccipital; **sq**, squamosal; **sqpf**, posterior flange of squamosal; **t**, tabular; **v**, vomer. Scale bar equals 1 cm.

**Fig 4:**
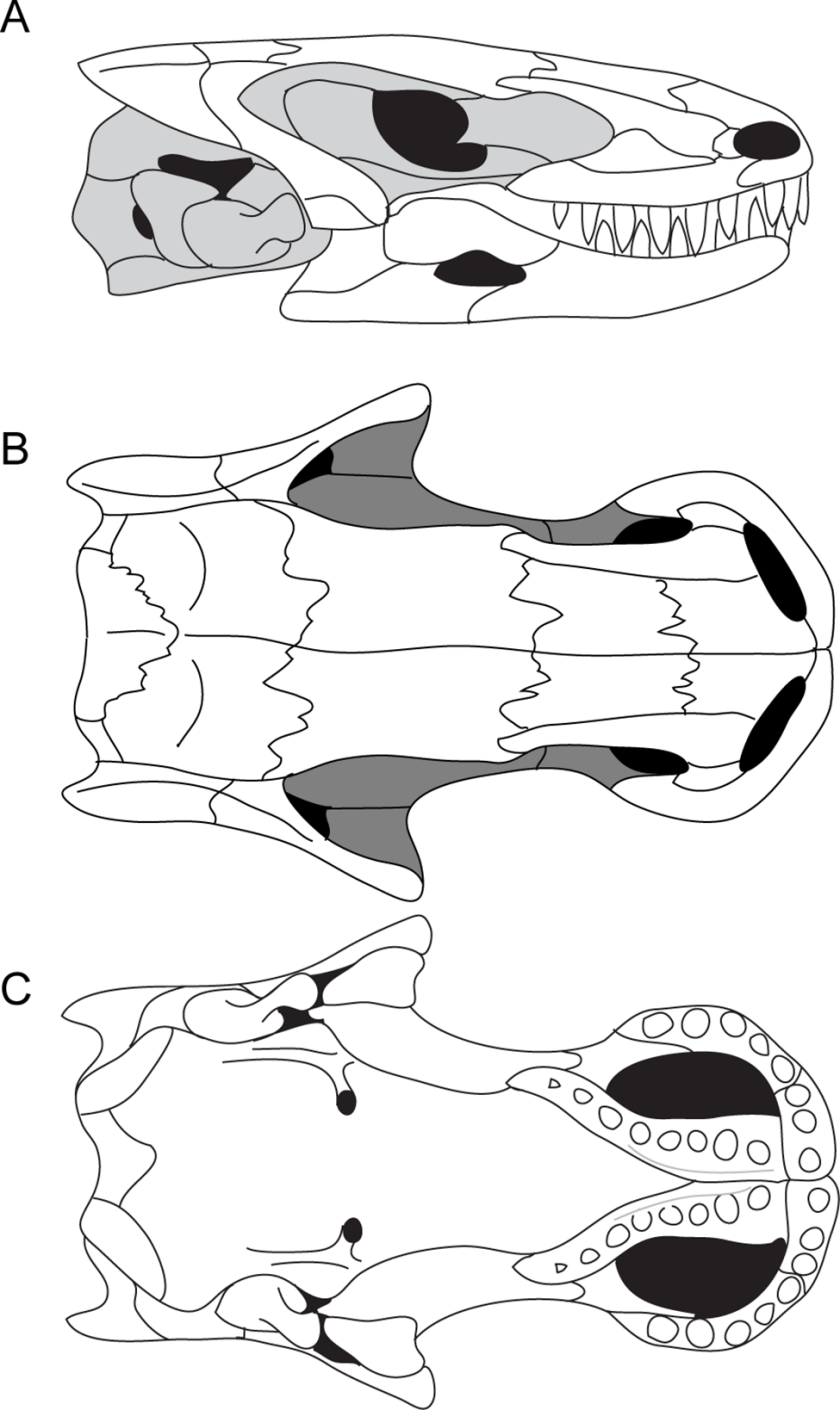
Reconstruction of the skull of *Brachydectes newberryi*. **A**, lateral view; **B**, dorsal view; **C**, palatal view. Image not to scale.

The skull of *Brachydectes newberryi* is extremely derived in comparison with that of other early tetrapods (including other lepospondyls) and exhibits significant reduction of dermal skull elements, especially along the orbit and cheek (Figs 3C and 4). Most strikingly, the lateral region of the cheek lacks dermal ossification between the orbit and the squamosal, resulting in a deep lateral emargination of the ventral cheek that is continuous with the orbit and extends dorsally to the parietals (Fig 3C). Previous workers have disagreed on the extent of the orbit within this region, and most recently Marjanovic and Laurin [16] suggested that the orbit had expanded to encompasse the entire lateral unossified regionto accommodate adductor musculature (although this was subsequently amended [17]). In the Council Grove skulls, a low postorbital process of the parietal is present near the prefrontal-parietal suture, confirming that the open cheek is formed through the loss of the postorbital bar, and that the orbit is very small and restricted to the very anterior extent of this unossified region (Fig 3). The remainder appears to be a greatly enlarged lateral cheek emargination, similar to that seen in the hapsidopareiontids *Hapsidopareion* and *Llistrofus* [32, 68, 69], the ostodolepids *Tambaroter* and *Pelodosotis* [32, 68, 70], and juveniles of the gymnarthrid *Cardiocephalus* (JSA, pers obs), although significantly deeper than seen in these taxa. This emargination may have accommodated enlarged adductor mandibulae externus musculature, in which case the morphology seen in *B*. *newberryi* may indicate further increase in the size of this muscle in *Brachydectes* in comparison with other lepospondyls.

The skull roof of *B*. *newberryi* was well described by previous authors [26, 28]. The median skull roof consists of paired nasals, frontals, parietals, and postparietals, as in the majority of early tetrapods. The nasals, frontals, parietals, and postparietals are all subequal in length. The prefrontal is well integrated into the anterior skull roof, forming a bridge between the frontals and maxillae, whereas posteriorly the tabular is reduced and integrated into the dermatocranial support for the suspensorium.

Anteriorly the nasals contact the premaxillae along a broad suture. Lateral to this is a broad, laterally-flaring emargination associated with the dorsal margin of the external naris. Posteriorly, the nasals overlap the frontals in a deep interdigitating suture. The median suture between the nasal pair is a straight butt joint without significant interdigitation.

The frontal is roughly rectangular, and is flanked laterally by the prefrontal, which excludes it from both the orbit, and the temporal emargination. The median suture between the frontal pair is straight but shows tongue-in-groove interdigitation in transverse section in larger specimens. Posteriorly, the frontal is sutured to the parietal via a series of deep lappets that incise posteriorly into the parietal. Some variation exists in the general shape of the frontal pair. In some specimens, the frontal pair is roughly rectangular, whereas in others the frontal pair is trapezoidal and tapers anteriorly. In a few specimens, the frontal pair expands anteriorly. The parietal pair is also broadly rectangular, and together the two parietals are as wide as the prefrontals and frontals combined. No pineal foramen is present and no pineal fossa is present on the ventral surface of the parietals. The median suture between parietals is essentially straight, with minimal interdigitation. Posteriorly, the parietal interdigitates with deep incisures in the postparietal pair.

The postparietals lie directly posterior, and are equal in width, to the parietals. The postparietals contribute to the sloping dorsal portion of the occiput, with a deep fossa on the occipital surface of each postparietal accommodating the epaxial musculature. No posttemporal fenestra is present in the occiput between the postparietals, the occipital arch, and the otic bones. The ventral surface of the postparietals contains impressions of the semicircular canals and may represent investment of the dorsal otic capsule by the postparietals.

The temporal region consists of two bones: the squamosal and the tabular (Fig 3). The squamosal makes up the majority of this region. It is a slender bone that descends from the occiput anteriorly towards the jaw articulation, intersecting the coronal plane at the level of the palate at an angle of approximately 40 degrees. The ventral end of the squamosal wraps around the lateral surface of the quadrate. A strong ridge is present on the occipital surface of the squamosal, and likely served as the point of origin of the depressor mandibulae.

The tabular is a small triangular bone that overlaps the dorsal portion of the squamosal and the lateral surface of the postparietal (Figs 2 and 3). The lateral surface of the bone is marked by a large fossa that appears to be an extension of the lateral emargination of the cheek. Posteriorly, the tabular projects beyond the occipital surface. This structure is not equivalent to the “tabular horns” seen in some nectrideans or the tabular prongs seen in adelospondyls. The posterior projection of the tabular forms the margin of a fossa for the epaxial musculature rather than constituting an actual projection from the posterior skull roof.

### Upper Dental Arcade

The premaxilla is small with a reduced pars dorsalis (Fig 3). No vomerine shelf of the premaxilla is present. The premaxillary dentition is reduced, with only three to four large, conical teeth. The pars dorsalis is aligned along the midline without an internarial fontanelle. The external nares are large, face anteriorly, and restrict the contact between the premaxilla and nasal to a small suture along the midline.

The maxilla is also reduced (Fig 3). No palatal shelf of the maxilla is present, but a tight suture does exist between the maxilla and palatine. The maxillary dentition is relatively reduced, with five to eight large conical teeth. The maxilla makes up the ventral margin of the orbit, but does not extend posterior to the postorbital process of the skull roof. The pars dorsalis is relatively limited, but is tightly sutured to the lacrimal in the antorbital region. Anteriorly, the maxilla participates in the margin of the external naris and choana.

### Braincase

**Fig 5:**
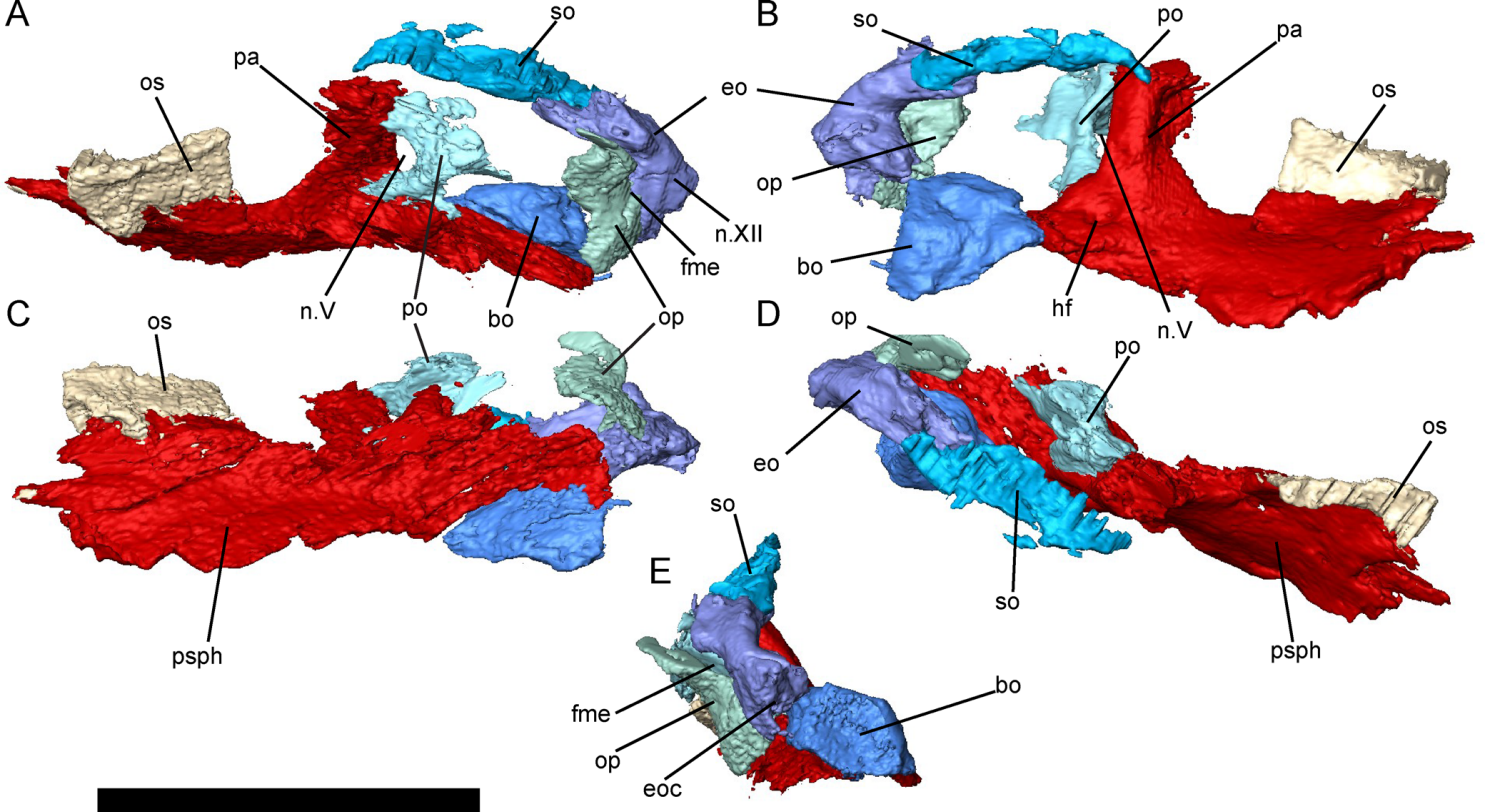
Braincase morphology of *Brachydectes newberryi*, DMNH 521121, 3D renders segmented from μCT. **A**, left lateral view; **B**, right medial view; **C**, ventral view; **D**, dorsal view; **E**, posterior view. Scale bar equals 5 mm. Abbreviations: **bo**, basioccipital; **eo**, exoccipital; **eoc**, exoccipital condyle; **fme**, foramen metoticum; **n.V**, foramen serving the trigeminal nerve (undivided); **n.XII**, foramen serving the hypoglossal nerve; **op**, opisthotic; **os**, orbitosphenoid; **pa**, pila antotica; **po**, prootic; **psph**, parasphenoid; **so**, supraoccipital.

**Fig 6:**
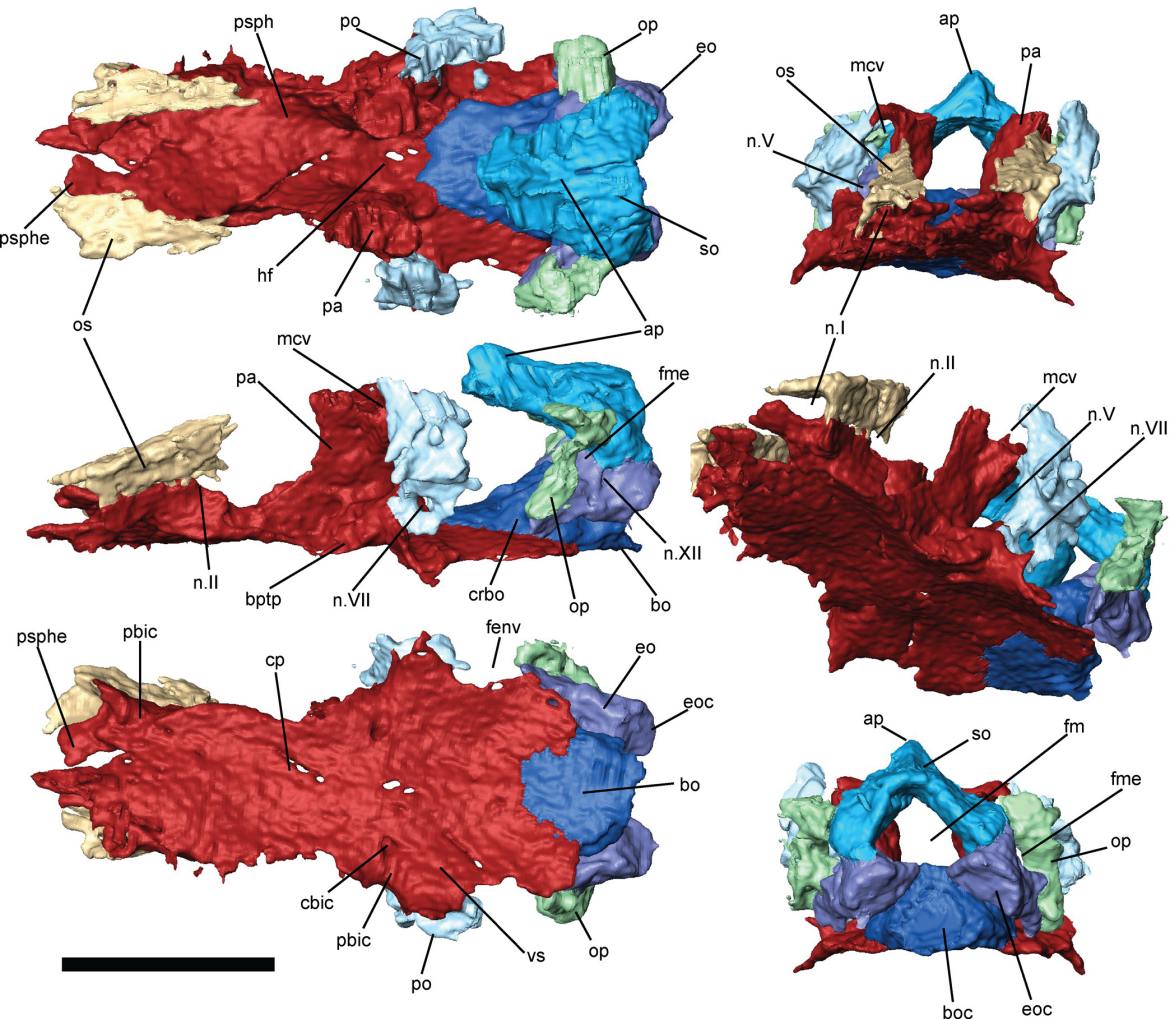
Braincase of *Brachydectes newberryi*, KUVP 49541, 3D renders segmented from μCT. **A**, dorsal view; **B**, anterior view; **C**, left lateral view; **D**, left anterolateral view; **E**, ventral view; **F**, occipital view. Scale bar equals 5 mm. Abbreviations: **ap**, median ascending process of the supraoccipital; **bo**, basioccipital; **boc**, basioccipital cotyle; **bptp**, basipterygoid process of parasphenoid; **cbic**, path of the cerebral branch of the internal carotid artery; **cp**, cultriform process of the parasphenoid; **crbo**, conical recess of the basioccipital; **eo**, exoccipital; **eoc**, exoccipital condyle; **fenv**, fenestra vestibularis; **fm**, foramen magnum; **fme**, foramen metoticum; **hf**, hypophyseal fossa; **mcv**, foramen serving middle cerebral vein; **n.I**, foramen serving olfactory nerve; **n.II**, foramen serving optic nerve; **n.V**, foramen serving trigeminal nerve (undivided); **n.VII**, foramen serving facial nerve; **n.XII**, foramen serving hypoglossal nerve; **op**, opisthotic; **os**, orbitosphenoid; **pa**, pila antotica; **pbic**, sulci serving the palatal branch of the internal carotid artery; **po**, prootic; **psph**, parasphenoid; **psphe**, presphenoid; **so**, supraoccipital; **vs**, vidian sulcus.

**Fig 7:**
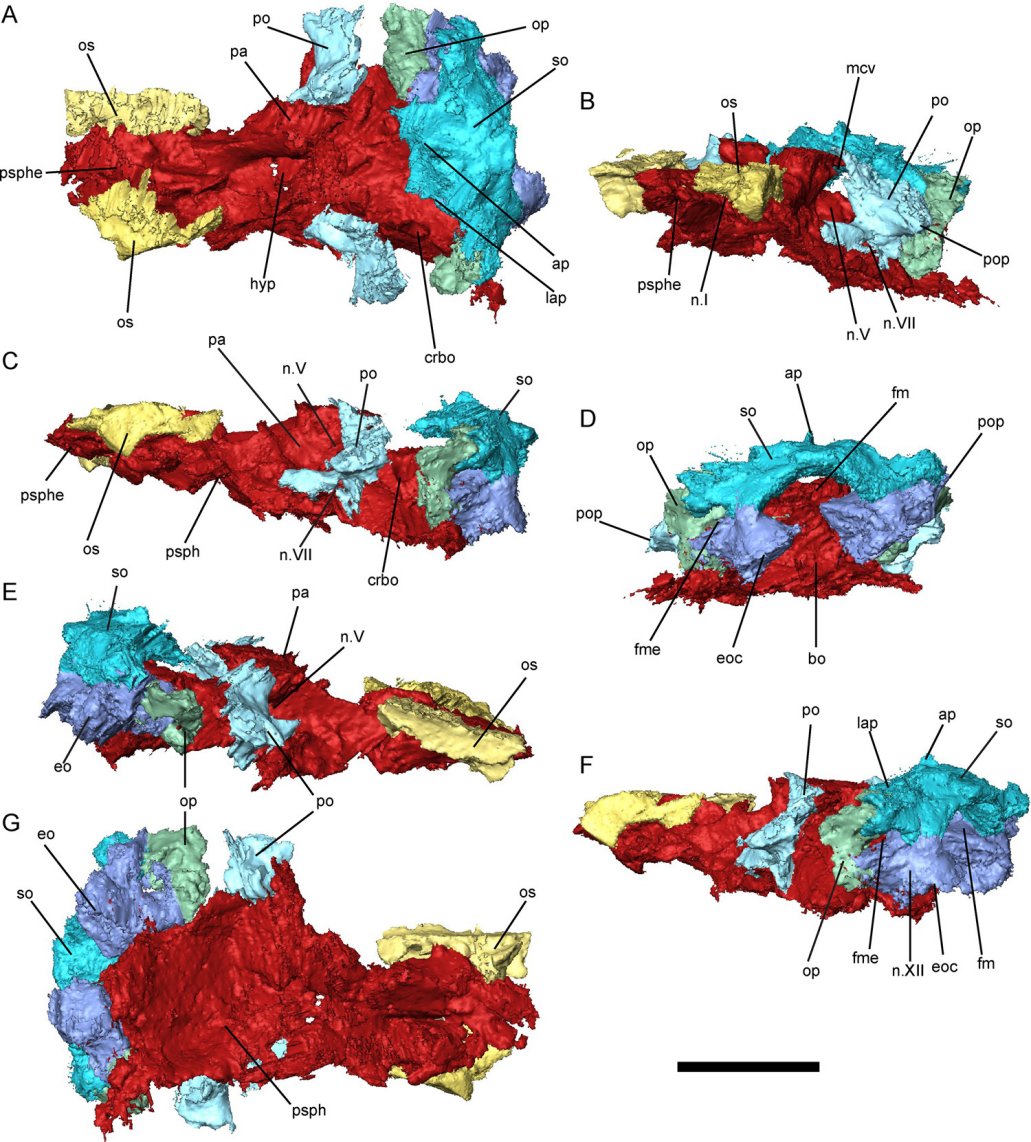
Braincase morphology of *Bracydectes newberryi* UNSM 32149, 3D renders segmented from μCT. **A**, dorsal view; **B**, left anterolateral view; **C**, left lateral view; **D**, occipital view; **E**, right lateral view; **F**, left posterolateral view; **G**, ventral view. Scale bar equals 5 mm. Abbreviations: **ap**, median ascending process of the supraoccipital; **bo**, basioccipital; **crbo**, conical recess of the basioccipital; **eo**, exoccipital; **eoc**, exoccipital condyle; **fm**, foramen magnum; **fme**, foramen metoticum; **hf**, hypophyseal fossa; **lap**, lateral ascending process of the supraoccipital; **mcv**, foramen serving the middle cerebral vein; **n.I**, foramen serving the olfactory nerve; **n.V**, foramen serving the trigeminal nerve (undivided); **n.VII**, foramen serving facial nerve; **n.XIII**, foramen serving hypoglossal nerve; **op**, opisthotic; **os**, orbitosphenoid; **pa**, pila antotica; **po**, prootic; **pop**, paroccipital process; **psph**, parasphenoid; **psphe**, presphenoid; **so**, supraoccipital.

The braincase of *B*. *newberryi* is well-ossified and robust (Figs 5–7). The orbitosphenoids, prootics, opisthotics, supraoccipital, exoccipitals, and basioccipital are all present as distinct elements, whereas regions of the braincase associated with the presphenoid, basisphenoid, and pila antotica are co-ossified with the parasphenoid.

The presphenoid (Figs 6 and 7) is a lozenge-shaped element at the base of the anterior region of the braincase. It is closely associated with the cultriform process of the parasphenoid, and these two elements may, in fact, be co-ossified, as no separate presphenoid ossification has been identified by previous workers [25, 26]. Dorsally, the presphenoid invades the columella ethmoidalis (Figs 6 and 7). Ossification of the columella ethmoidalis fills the entire space between the olfactory nerve foramina in the largest specimen studied (Fig 7) but is restricted to the areas surrounding the olfactory nerves in smaller specimens (Figs 5 and 6) and absent entirely in the smallest specimens. Otherwise, the presphenoid is dorsoventrally flattened along its length and does not form an interorbital septum, in contrast with the condition seen in brachystelechids [41, 43] but consistent with the morphology seen in a number of other recumbirostrans [33, 42, 71]. A presphenoid has previously been described in *Carrolla craddocki* as the sphenethmoid [41] and appears to be present in a variety of other microsaurs as well [42, 43]. Paired orbitosphenoids make up the lateral wall of the anterior sphenoid region, extending from the presphenoid posteriorly to the optic foramen (Fig 6). The orbitosphenoid in *B*. *newberryi* is a long, low, robust vertical wall similar in morphology to that of *Carrolla craddocki* [41] and *Quasicaecilia texana* [43] rather than the thin, bulging structure seen in more conservative ‘microsaurs’ [33, 42]. A deep notch is present at the posteroventral corner of the bone for accommodation of the optic nerve (Figs 6 and 7). Unlike the condition seen in many recumbirostrans [33, 42], no descending flange of the frontal articulates with the orbitosphenoid. Anteriorly, the surface of the orbitosphenoid is smooth and lacking in facets for articulation with the trabecular plate dorsally and the antorbital cartilage ventrally, in contrast with the condition seen in lissamphibians [60]. It appears likely that no lateral connection between the orbitosphenoid and the nasal capsule was present in *Brachydectes*. Similar morphology has been observed in a number of other recumbirostrans [33, 41–43, 71], and this may represent a more general ‘microsaur’ condition. The pila metoptica is unossified, consistent with the morphology found in *Carrolla craddocki* [41], *Quasicaecilia texana* [43], *Huskerpeton englehorni* [33], and *Rhynchonkos stovalli* [42].

The pila antotica is fully ossified, tall, robust, teardrop-shaped in cross section, and firmly sutured to the ventral surface of the parietal (Figs 5–7). Between the pilae antoticae, the hypophyseal fossa is broad and weakly posteriorly-directed (Fig 6). The dorsum sellae delineates the posterior margin of the shallow hypophyseal fossa at the midline, but ascend laterally into broad ridges along the medial surface of the pilae antoticae, and reach the anterior contact between the pilae antoticae and the parietals.

Posterior to the pila antotica, there is a large antotic fenestra (Figs 5–7), which in larger specimens is partially to completely bisected by a bony bridge into a dorsal and ventral foramen. Anteriorly, the dorsal foramen opens into a sulcus on the ventral surface of the parietals, and posteriorly passes dorsally and medially to the semicircular canals of the inner ear (Fig 7, sov). The ventral foramen has been identified by previous authors as serving the postganglionic trunk of the trigeminal nerve [41–43], whereas the dorsal foramen has generally been interpreted as serving a vein [41, 43], likely the middle cerebral vein. Both fenestrae are very large in comparison with other recumbirostrans, whereas in other recumbirostrans these foramina are of moderate size [41–43].

**Fig 8:**
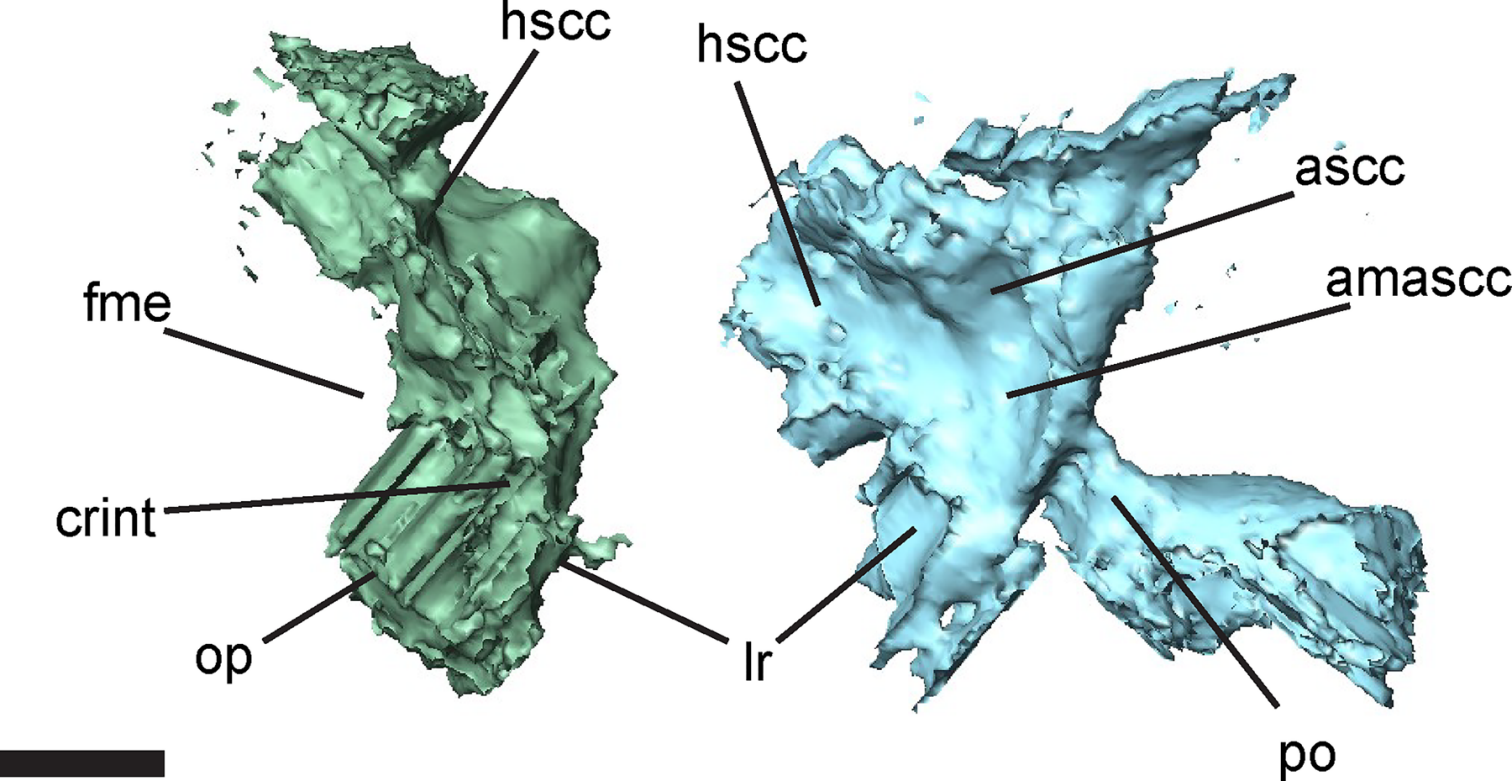
Selected otic elements of the left otic capsule of *Brachydectes newberryi*, UNSM 32149, in medial view. Scale bar equals 1 mm. Abbreviations: **amascc**, fossa enclosing ampulla of the anterior semicircular canal; **ascc**, sulcus for anterior semicircular canal; **crint**, crista interfenestralis; **fme**, foramen metoticum; **hscc**, sulcus for horizontal semicircular canal; **lr**, lagenar recess; **op**, opisthotic; **po**, prootic.

The otic capsules consist of distinct prootic and opisthotic elements (Figs 5–7). In smaller specimens, the prootic and opisthotic are small and relatively distinct (Fig 5), but in the largest specimen (UNSM 32149) these elements are more completely ossified, and approach each other ventral to the horizontal semicircular canal (Fig 7). The prootic is roughly triangular, with the base sutured to the parasphenoid at the level of the basipterygoid processes. The opisthotic is tall and pillar-like, with a strong vertical crista along the medial surface, here interpreted as a crista interfenestralis (Fig 8). The prootic and opisthotic co-ossified with the squamosal and parietal dorsally and with the parasphenoid ventrally (Fig 7). The fenestra vestibularis is roughly oval in shape and fits the stapes closely both anteriorly and posteriorly, seemingly without space for an opercular cartilage. Laterally, the crista parotica exists as a strong horizontal ridge on the prootic, bracing against the squamosal. This ridge may be homologous to the paroccipital process of amniotes [72], although the latter involves more significant contributions from the opisthotic.

**Fig 9:**
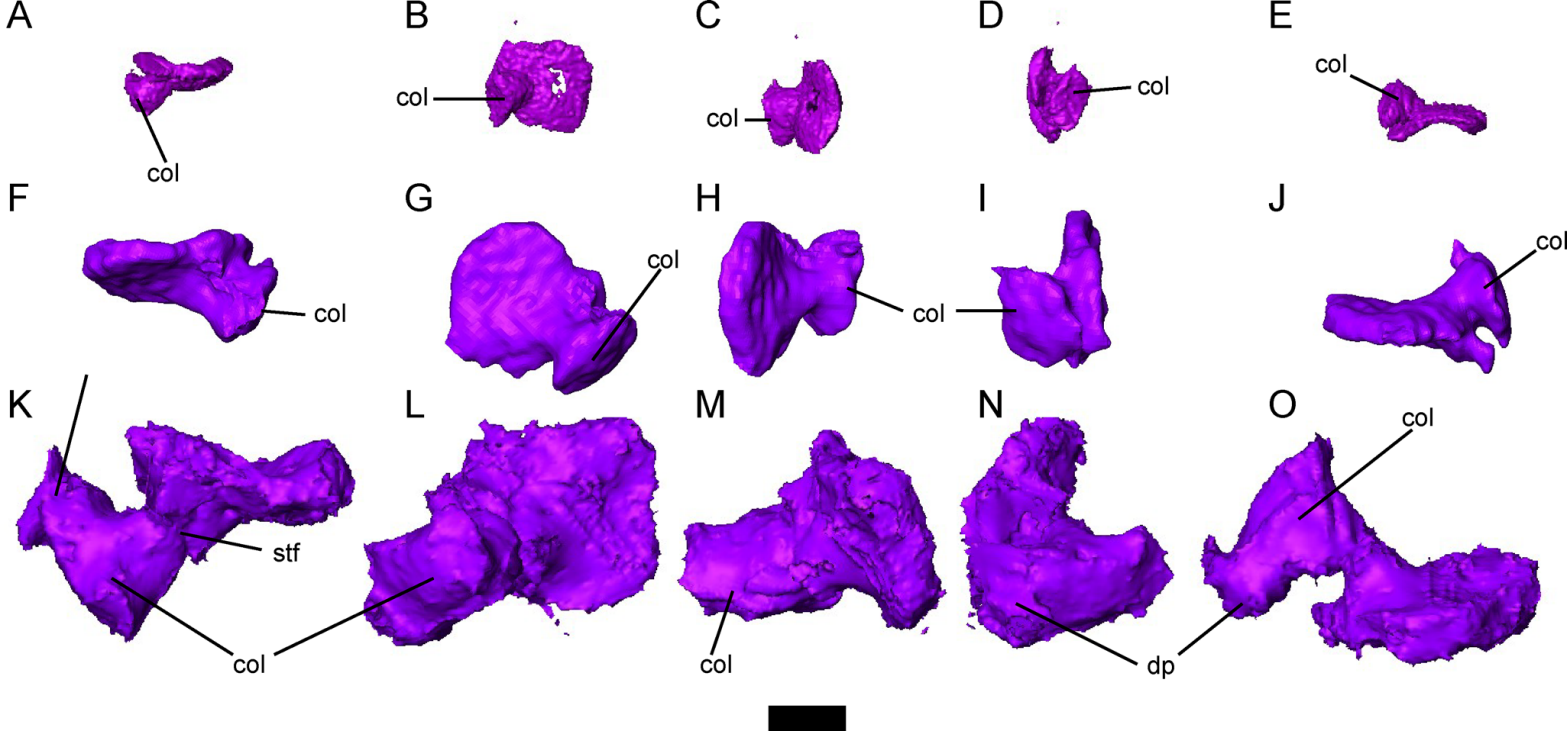
Morphology of the stapes in small, subadult, and skeletally mature *Brachydectes newberryi*. **A-E**, DMNH 521121, left stapes in **A**, dorsal; **B**, left lateral; **C**, posterior; **D**, anterior; and **E**, ventral view; **F-J**, KUVP 49541, right stapes in **F**, dorsal; **G**, right lateral; **H**, posterior; **I**, anterior; and **J**, ventral view; **K-O**, UNSM 32149, left stapes in **K**, dorsal; **L**, left lateral; **M**, posterior; **N**, anterior; and **O**, ventral view. Scale bar equals 1 mm. Abbreviations: **col**, columellar process of stapes; **dp**, dorsal process of columella of stapes; **stf**, stapedial foramen.

The stapes has a round to oval footplate and a well-developed columella, and is well-ossified in larger specimens (Fig 9). A shallow groove is present along the perimeter of the footplate. The columella flares distally, forming a broad articulation with the dorsal portion of the quadrate. A small foramen halfway along the length of the columella likely transmitted the stapedial artery (Fig 9). A robust dorsal process is present in the largest specimen (Fig 9K–9O), but not in smaller specimens (Fig 9A–9E), which extends proximally from the midpoint of the columella towards the crista parotica, although it does not directly articulate with the otic capsule. The deep notch between the dorsal process of the stapes and the base of the columella would likely have encased the lateral head vein. In some specimens, the columella is weakly mineralized between the footplate and the distal tip, and in some cases the distal tip of the columella appears to be a separate distinct ossification. It is possible that this element may be a distinct extracolumella (as is seen in some reptiles and amphibians), or that it represents a second ossification center in an element that co-ossifies in fully adult specimens.

The occipital arch consists of a well-ossified basioccipital, paired exoccipitals, and a single median supraoccipital (Figs 5–7). Surfaces on the basioccipital and exoccipitals contribute to the occipital cotyle, which is unpaired and crescentic. The occipital cotyle is weakly concave, and accepts the odontoid process of the atlas. The bases of the exoccipitals are swollen anteriorly along the suture with the parasphenoid. The metotic foramen passes through the suture between the exoccipital and opisthotic, and served as the passage for the vagus nerve and the jugular vein. A second foramen, which pierces the exoccipital just lateral to the occipital cotyle, served the hypoglossal nerve (Figs 6 and 7). The synotic tectum is occupied by a single broad supraoccipital bone, as in other ‘microsaurs’ [33, 42]. A pointed ascending process of this ossification extends anteriorly beneath the postparietals and is exposed medially up to the posterior margin of the parietals. Posttemporal fossae are fully closed. No supraoccipital sinus is present. A posterior shelf of the supraoccipital roofs the foramen magnum; the dorsolateral surfaces of this shelf serve as an articular surface for the proatlas. The supraoccipital is present as a single ossification across all ontogenetic stages studied here.

### Palate

The parasphenoid of *Brachydectes newberryi* is a broadly triangular element in ventral view, and represents the majority of the ossified palate. The cultriform process is laterally expanded to the extent that it is nearly as broad as the distance between the basipterygoid processes, and extends to the anterior end of the orbitosphenoid ossification, where it tapers rapidly to a point. The basal plate of the parasphenoid is roughly rectangular, without the broad triangular posterior expansion seen in some ‘microsaurs’ [33, 41–43]. The basipterygoid processes lie directly ventral to the pilae antoticae, and are together narrower than the widest extent of the basal plate of the parasphenoid. Posteriorly, the parasphenoid is deeply notched, exposing the basioccipital. The parasphenoid is firmly sutured to the ventral surface of both the anterior and posterior regions of the braincase.

The branches of the internal carotid artery leave clear grooves and canals on and through the ventral and lateral surface of the parasphenoid (Fig 6), recording the pattern of some of the cranial circulation in *Brachydectes*. The route of the internal carotid artery coursed along a sulcus on the ventral surface of the parasphenoid, just medial to the basipterygoid processes. At the level of the latter, this sulcus gives rise to another deep groove medially that terminates at a foramen that pierces the basal plate of the parasphenoid and emerges from the medial surface of the pleurosphenoid within the hypophyseal fossa (Fig 6E). This canal would have enclosed the cerebral branch of the internal carotid artery. The main sulcus continues laterally, anterior to the basipterygoid process, and follows a shallow groove on the lateral surface of the cultriform process. This groove would have housed the palatal branch of the internal carotid artery. In contrast to the condition seen in temnospondyls [73] and lissamphibians [74] where the internal carotid divides into cerebral and palatal branches either within the parasphenoid itself or within the hypophyseal fossa, the internal carotid artery of *Brachydectes* appears to have been divided into cerebral and palatal branches prior to entering the braincase, consistent with the morphology seen in some stem and crown amniotes [75].

**Fig 10:**
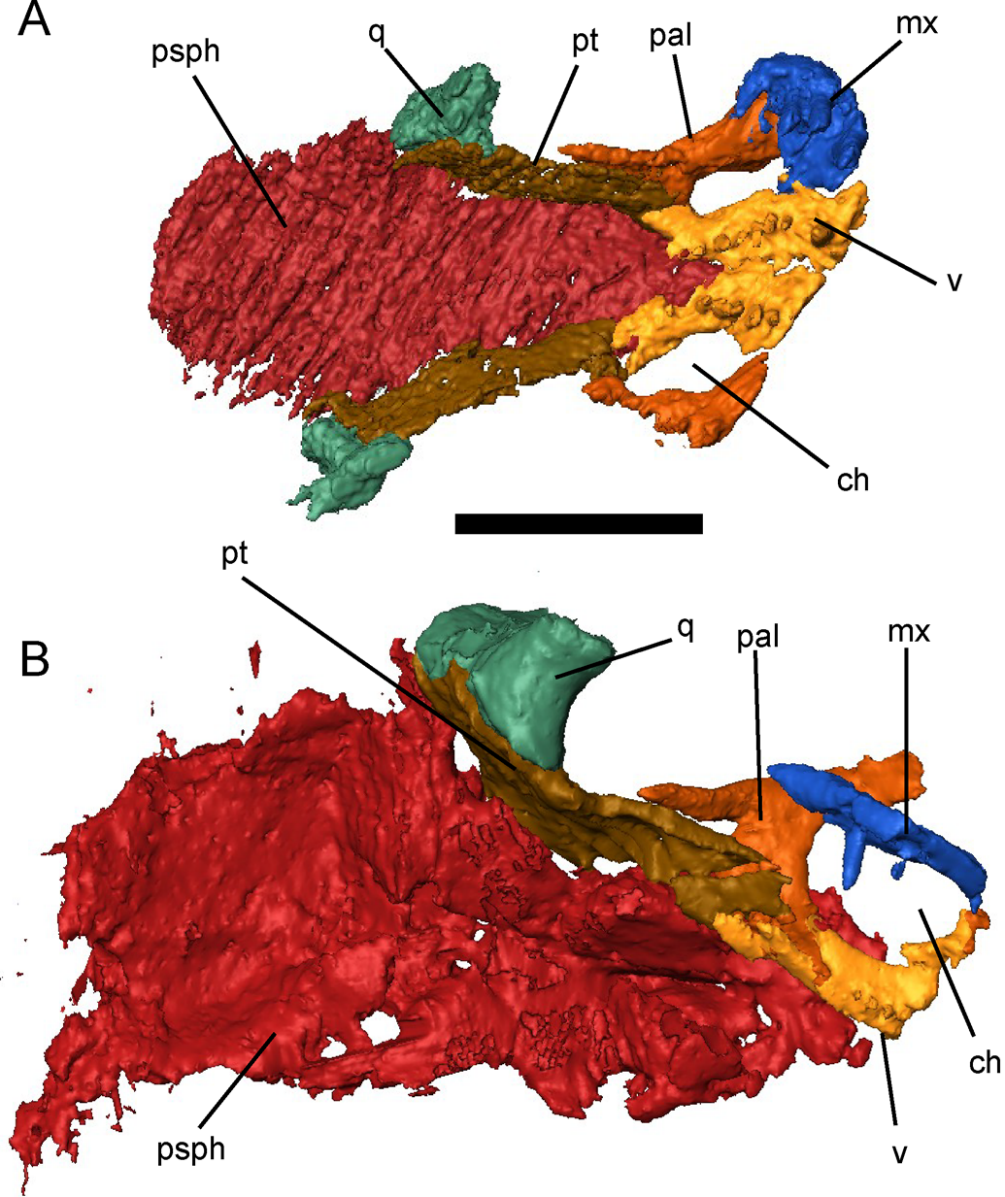
Selected skulls of *Brachydectes newberryi* in palatal view. **A**, KUVP 49539; **B**, UNSM 32149. Scale equals 5 mm. Abbreviations: **ch**, choana; **mx**, maxilla; **pal**, palatine; **psph**, parasphenoid; **pt**, palatine; **q**, quadrate; **v**, vomer.

The vomers are narrow, elongate bones that contact each other anteriorly at the midline of the palate, anterior to the cultriform process of the parasphenoid (Fig 10). A short premaxillary process extends along the midline of the anterior palate to meet the premaxilla, but neither a premaxillary shelf nor a maxillary process or shelf is present. Posteriorly, the palatine process of the vomer is narrow and extends just lateral to the cultriform process. A series of six to eight large, robust teeth extends along the midline of each vomer.

The palatine is a short, strutlike element that connects the maxilla to the palatine ramus of the pterygoid (Fig 10). The palatine and maxilla are typically found together in specimens even when disarticulated from the remainder of the skull, suggesting that these elements may be more tightly sutured than the remainder of the palate. Neither teeth nor denticles are present on the palatines.

**Fig 11:**
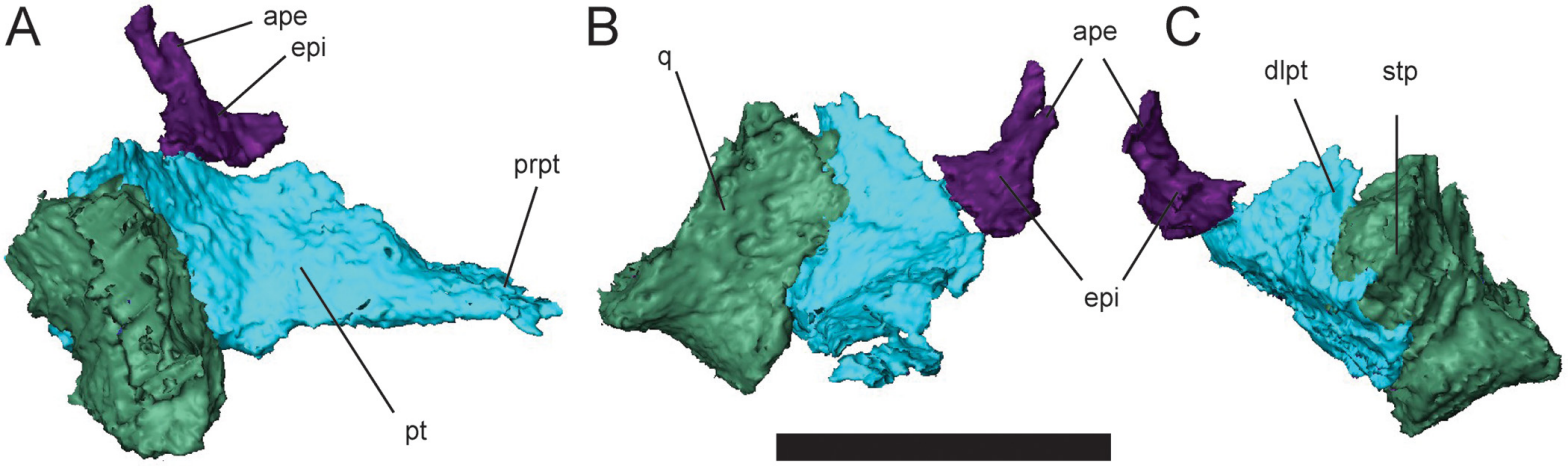
Right palatal and suspensorial elements of *Brachydectes newberryi*, UNSM 32149. **A**, lateral view; **B**, anterior view; **C**, posterior view. Scale equals 5 mm. Abbreviations: **ape**, ascending process of epipterygoid; **dlpt**, dorsal lamina of pterygoid; **epi**, epipterygoid; **prpt**, palatine ramus of pterygoid; **pt**, pterygoid; **stp**, stapedial process of quadrate.

The pterygoids are greatly reduced in comparison with those of other early tetrapods (Figs 3E, 10 and 11). The palatine ramus is foreshortened, but still retains a lateral contact with the palatine and an anterior contact with the vomers. The body of the pterygoid extends adjacent to the posterior portion of the cultriform process of the parasphenoid, eliminating any interpterygoid vacuity. The quadrate process is anteriorly displaced and descends obliquely along the medial surface of the quadrate. The medial surface of the pterygoid is reflected dorsally to form a low dorsal process lateral to the pila antotica. The basal process is reduced to a shallow facet at the anterior extremity of the dorsal process. No transverse process extends into the subtemporal vacuity. Teeth and denticles are absent from the body of the pterygoid.

An epipterygoid is present and ossified only in the largest specimen scanned, UNSM 32149 (Fig 11). The epipterygoid is a simple element, with a broad, roughly-horizontal ventral facet for articulation with the pterygoid, and a simple, slender dorsal stalk, comparable to the condition seen in amniotes. The epipterygoid does not articulate with the skull roof and appears to serve a reduced role in support of the palate in comparison with the recumbirostrans *Pantylus cordatus* [76], *Huskerpeton englehorni* [33], *Rhynchonkos stovalli* [42], *Aletrimyti gaskillae* [42], and *Dvellecanus carrolli* [42]. The epipterygoid contributes substantially to the conus recessus.

The quadrate is relatively simple in morphology (Fig 11). The condyle is trochlear and is somewhat mediolaterally compressed. The anterior surface of the quadrate is weakly concave, with a weak transverse ridge running from the lateral trochlea of the condylar surface to the medial surface. Posteriorly the quadrate tapers to a sharp ridge, which extends to a tubercle-like stapedial process in the largest specimen. A shallow sulcus present on the medial surface may be equivalent to the sulcus assigned to the so-called “chorda tympani” nerve.

### Lower Jaw

**Fig 12:**
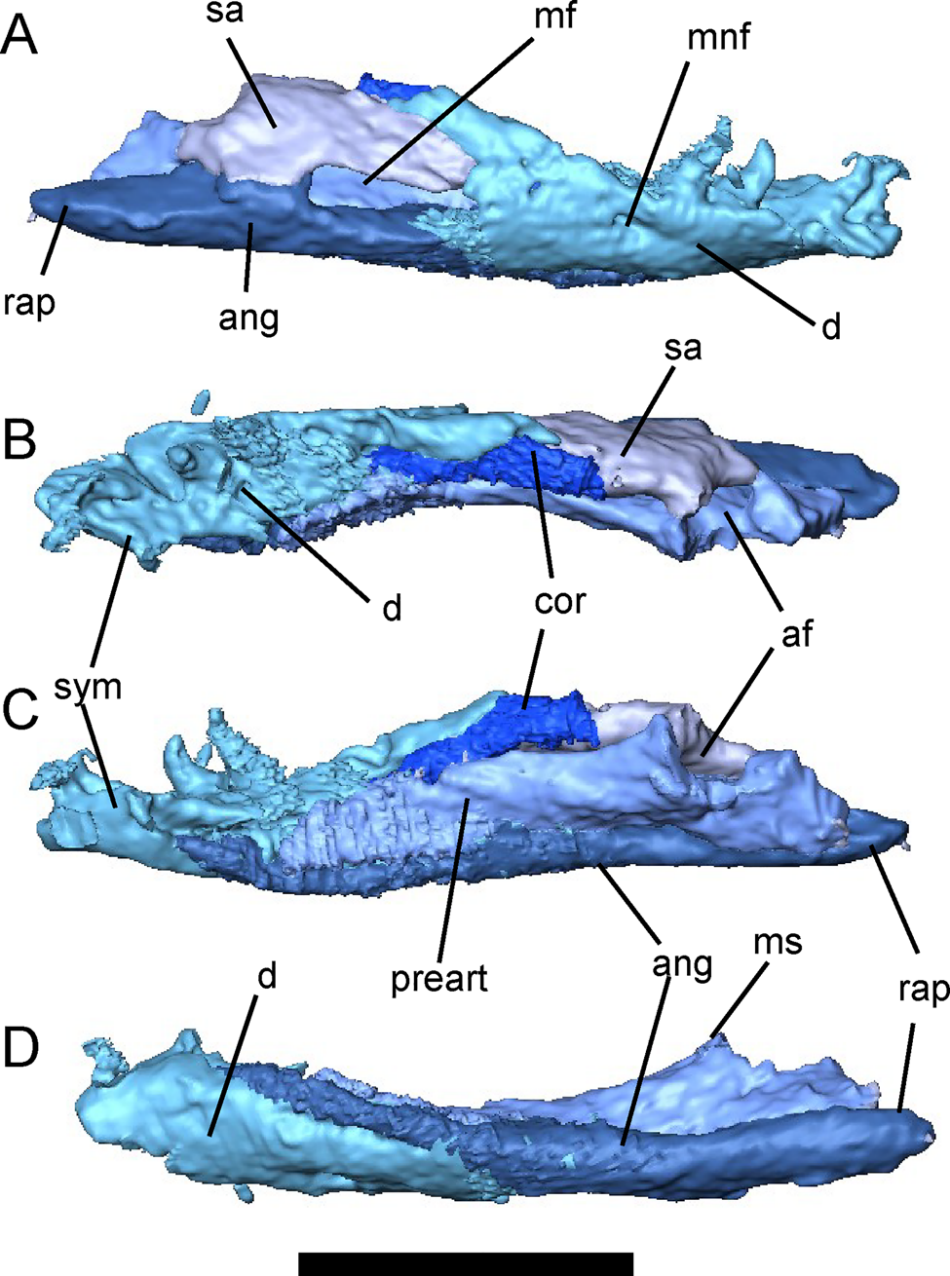
Right lower jaw of *Brachydectes newberryi*, KUVP 49541, rendered from μCT. **A**, lateral view; **B**, dorsal view; **C**, medial view; **D**, ventral view. Scale equals 5 mm. Abbreviations: **af**, articular fossa; **ang**, angular; **cor**, coronoid; **d**, dentary; **mf**, mandibular fenestra; **mnf**, mental foramen; **ms**, medial shelf of prearticular; **rap**, retroarticular process; **sa**, surangular; **sym**, mandibular symphysis.

The lower jaw of *Brachydectes newberryi* has been adequately described by previous workers [25, 28] and little difference exists between the described morphology and that observed in the Council Grove skulls (Fig 12). The number of ossifications is greatly reduced from the basal tetrapod complement; only a dentary, angular, surangular, prearticular, and coronoid are present. The dentary is relatively short with a deep coronoid expansion that is continued posteriorly by the surangular. As previously described, *Brachydectes newberryi* has a relatively low tooth count, with approximately five to six teeth. The dentary is the primary bone of the lower jaw, and forms, with the coronoid, a large well-developed coronoid process. It appears to be the only bone contributing to the symphysis. A single, small Meckelian foramen pierces the dentary around the posterior extent of the tooth row. The coronoid is simple, with a small anterior process, but not precluding extensive contact between the dentary and prearticular. Teeth and denticles are both absent on the coronoid. The prearticular makes up the majority of the medial surface of the jaw. The articular is co-ossified with the prearticular and is narrow but deep, fitting closely into the trochlea of the quadrate. Posterior to the articular fossa, the angular forms an elongate, straight retroarticular process with a median contribution from the articular. A large mandibular fenestra is present laterally, between the dentary, angular and surangular.

### Cranial Endocast

**Fig 13:**
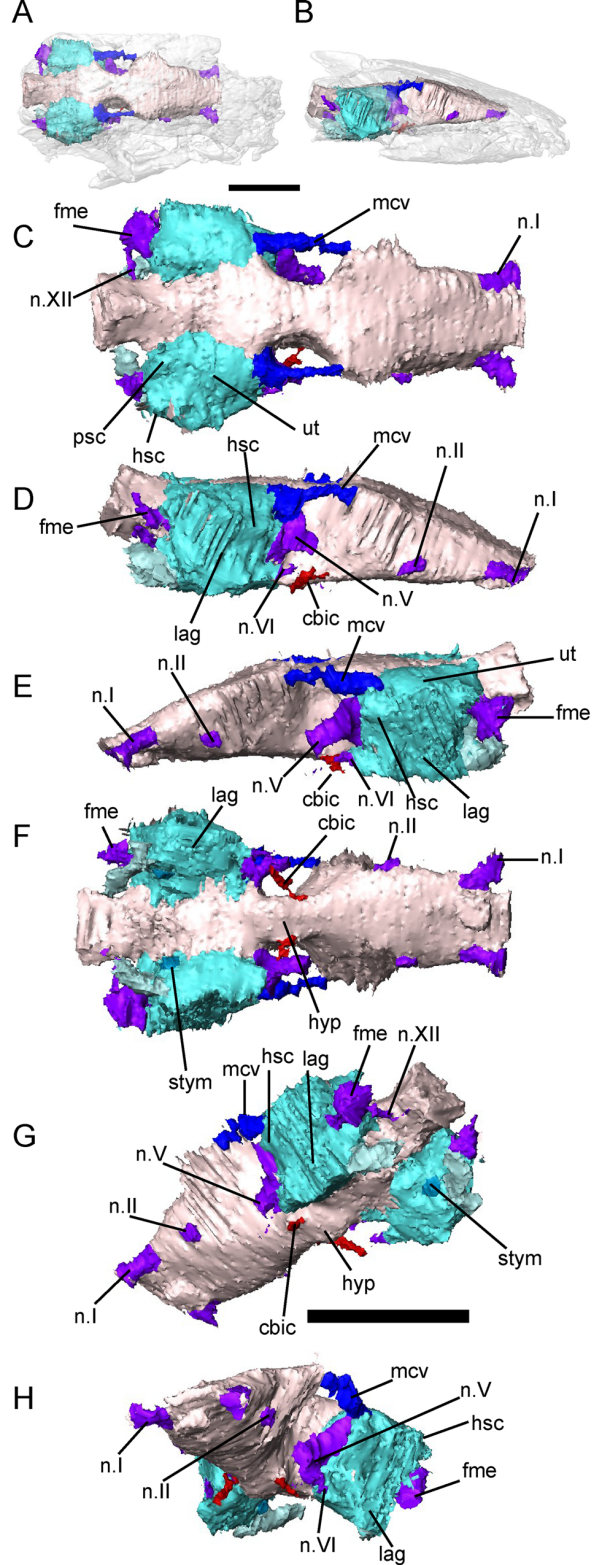
Cranial endocast of *Brachydectes newberryi*, KUVP 49541, rendered from μCT. **A-B**, position of cranial endocast within complete skull, **A**, dorsal view; **B**, lateral view; **C**, endocast in dorsal view; **D**, endocast in right lateral view; **E**, endocast in left lateral view; **F**, endocast in ventral view; **G**, endocast in endocast in left posteroventral oblique view; **H**, left anteroventral oblique view. Scale equals 5 mm. Abbreviations: **cbic**, passage of the cerebral branch of the internal carotid artery; **fme**, passage of the foramen metopticum; **hsc**, horizontal semicircular canal; **hyp**, hypophyseal fossa; **lag**, fossa enclosing lagena; **mcv**, passage serving middle cerebral vein; **n.I**, passage of the olfactory nerve; **n.II**, passage of the optic nerve; **n.V**, passage of the trigeminal nerve; **n.VI**, passage of the facial nerve; **n.XII**, passage of the hypoglossal nerve; **psc**, posterior semicircular canal;; **stym**, fossa possibly accommodating scala tympani; **ut**, utricular fossa.

In order to better characterize the morphology of the cavum cranii and otic capsule, we produced a virtual endocast of KUVP 49541 (Fig 13). The cavum cranii is roughly rectangular in dorsoventral view, and wedge-shaped in lateral view, without significant curvature of the AP axis. The cavum cranii is expanded anteriorly, constricted in the region of the hypophysis, then expands again posteriorly.

The telencephalon region of the endocast, enclosed laterally by the orbitosphenoids, contains the olfactory bulbs and cerebrum. The canals enclosing the olfactory nerves are widely-spaced and diverge anteriorly. Unlike the condition seen in some recumbirostrans [33, 42], there are not distinct fossae accommodating the olfactory lobes and cerebral hemispheres, but the passage for the optic nerve serves as an absolute posteriormost limit on the size of the olfactory bulbs. The passage for the optic nerve is situated at the bottom of the ventral surface of the forebrain region, approximately halfway between the hypophyseal fossa and the passages for the olfactory nerves.

The diencephalon, enclosed laterally by the ossified pila antotica, appears as a marked medial constriction between the cerebrum and otic capsules. Ventrally there is a shallow expansion of the endocast accommodating the hypophysis. The passages for the cerebral branches of the internal carotid artery converge anteriorly to meet the hypophysis. Dorsally, neither a canal nor a fossa is present to accommodate the epiphysis, suggesting that this structure may have been highly reduced in comparison with other early tetrapods.

The medial wall of the otic capsule is largely unossified, preventing clear delineation of structures in the midbrain. However, the endocast does expand dorsally and somewhat laterally posterior to the pila antotica. This expansion of the endocast may accommodate the cerebellum and optic tectum, or cerebellum alone, depending on the organization of the diencephalon and midbrain. A canal, possibly serving the middle cerebral vein, meets the endocast lateral to this expansion, passing between it and the ampulla of the anterior semicircular canal of the otic capsule. The canal serving the trigeminal emerges ventral to this canal, just posterodorsal of the hypophyseal fossa. A small canal lateral to this and entering the endocast ventromedial to the space accommodating the lagena may represent the course of the facial nerve.

Posterior to the otic capsules, the dorsal surface of the endocast is arched, producing an endocast that is roughly triangular in transverse section. Two canals are present in the hindbrain region: a large canal representing the path of the metotic foramen and a smaller canal representing the path of the hypoglossal nerve. The metotic canal, which would have enclosed the glossopharyngeal, vagus, and accessory nerves as well as the internal jugular vein, meets the endocast ventral to the posterior semicircular canal. The canal serving the hypoglossal nerve meets the hindbrain region of the endocast posterior to the metotic canal.

**Fig 14:**
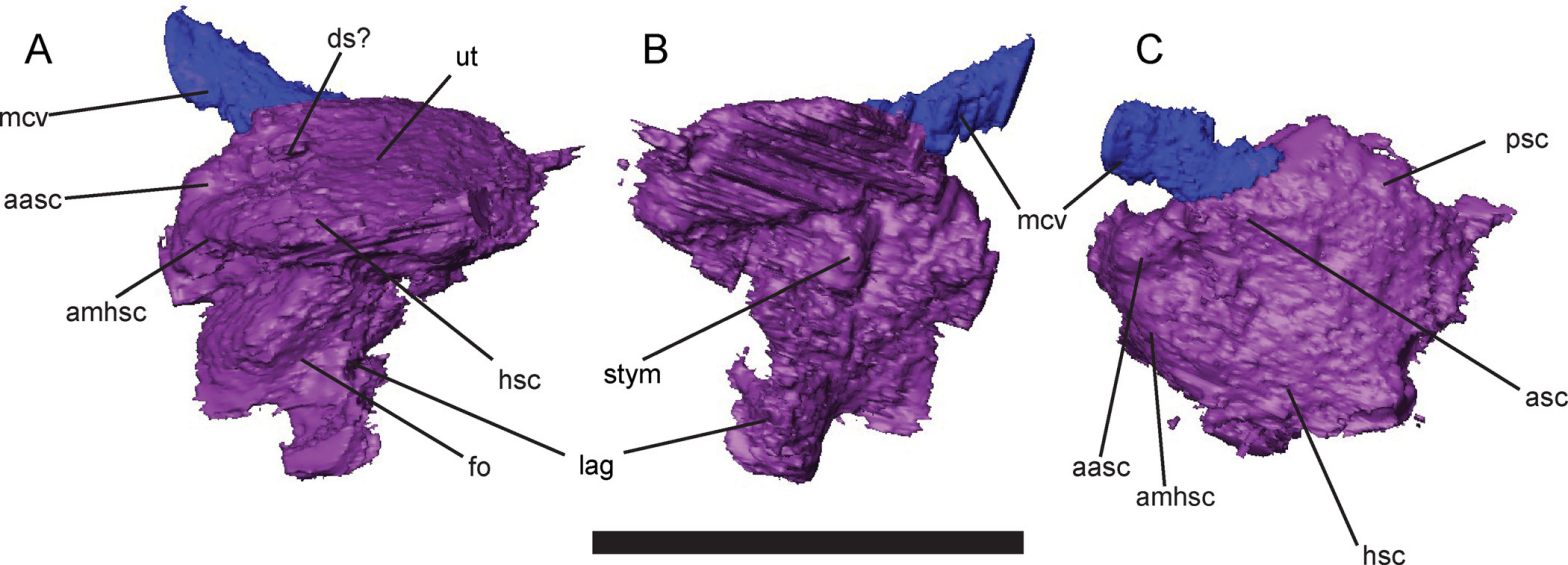
Left otic endocast of *Brachydectes newberryi*, KUVP 49541, rendered from μCT. **A**, left lateral view; **B**, medial view; **C**, dorsal view. Scale equals 5 mm. Abbreviations: **aasc**, ampulla of the anterior semicircular canal; **amhsc**, ampulla of horizontal semicircular canal; **asc**, anterior semicircular canal; **ds**, dorsal sulcus; **fo**, surface of fenestra vestibularis; **hsc**, horizontal semicircular canal; **lag**, fossa accommodating lagena; **mcv**, passage of middle cerebral vein; **psc**, posterior semicircular canal; **stym**, fossa possibly accommodating the scala tympani,

The medial surface of the otic capsule of UNSM 32149 preserves osteological correlates of the structure of the inner ear, permitting creation of a virtual endocast of the inner ear and description of the gross morphology of this structure in greater detail (Fig 14). Traces of the courses of the semicircular canals and saccular region are preserved as sulci along the medial surface of the otic bones. The semicircular canals are restricted to the upper portion of the otic capsule and directly abut the utriculus, with no intervening bone. Ampullae are preserved as weak swellings in the fossae for the semicircular canals, but lack distinct morphology. The horizontal semicircular canal is laterally exposed just ventral to the crista parotica. A large saccular region is present ventral to the utriculus, and is expanded greatly below the semicircular canals. A medial projection of the saccular region is preserved as a pit in the basioccipital bone, and may have housed the scala tympani (Fig 14). The well-developed crista interfenestralis forms a solid ossified barrier between the inner ear and metotic fossa and precludes a posterior path of the re-entrant fluid circuit. The medial wall of the otic capsule is incompletely ossified, preventing detailed characterization of the paths of the facial, vestibulocochlear, and abducens nerves.

## Discussion

### Affinities of *Brachydectes*

The phylogenetic relationships of lysorophians have generally been considered in terms of three main issues: the relationship between lysorophians and modern lissamphibians, the relationship between lysorophians and other elongate-bodied lepospondyls, and the relationship between lysorophians and ‘microsaurs.’ The endocranial data presented here suggests a resolution to these problems may be attainable, although an expansive phylogenetic analysis is outside the scope of this study and will appear elsewhere.

#### *Brachydectes* and lissamphibian origins

Differences between the braincases of *Brachydectes* and of lissamphibians are conspicuous and substantive. Major differences include: the composition of the occipital arch, structure of the occipital condyles, site of insertion of the hypaxial musculature on the ventral braincase, the course of the internal carotid and its branches, the course of the perilymphatic circulation, the course of the lateral head vein, and the structure of the cartilages of the ethmoid trabeculae.

The occipital arch of *Brachydectes newberryi* consists of paired exoccipitals, a median ventral basioccipital, and a definitive amniote supraoccipital that compares well with the morphology described for the eureptiles *Captorhinus* [72], *Petrolacosaurus* [77], and *Youngina* [78]. In contrast, the occipital arch of lissamphibians consists of only paired exoccipitals, which in some species invade the synotic tectum and basioccipital region partially or completely [79]. No basioccipital or supraoccipital element is present in any lissamphibian, fossil or modern. The presence of a basioccipital in *Brachydectes* is plesiomorphic for tetrapods and thus phylogenetically uninformative, but the presence of a well-developed median supraoccipital is restricted to the amniote crown [80] and recumbirostran ‘microsaurs’. Although the supraoccipital of *Brachydectes* and ‘microsaurs’ has traditionally been considered convergent with the amniote supraoccipital, new data from μCT have demonstrated that the ‘microsaur’ supraoccipital shares a number of morphological details with early amniotes, and early eureptiles in particular, and is likely homologous with the amniote element [42]. This homology does not extend far down the amniote stem, as seymouriamorphs lack a supraoccipital and ‘anthracosaurs’ generally exhibit paired elements within the synotic tectum [80].

**Fig 15:**
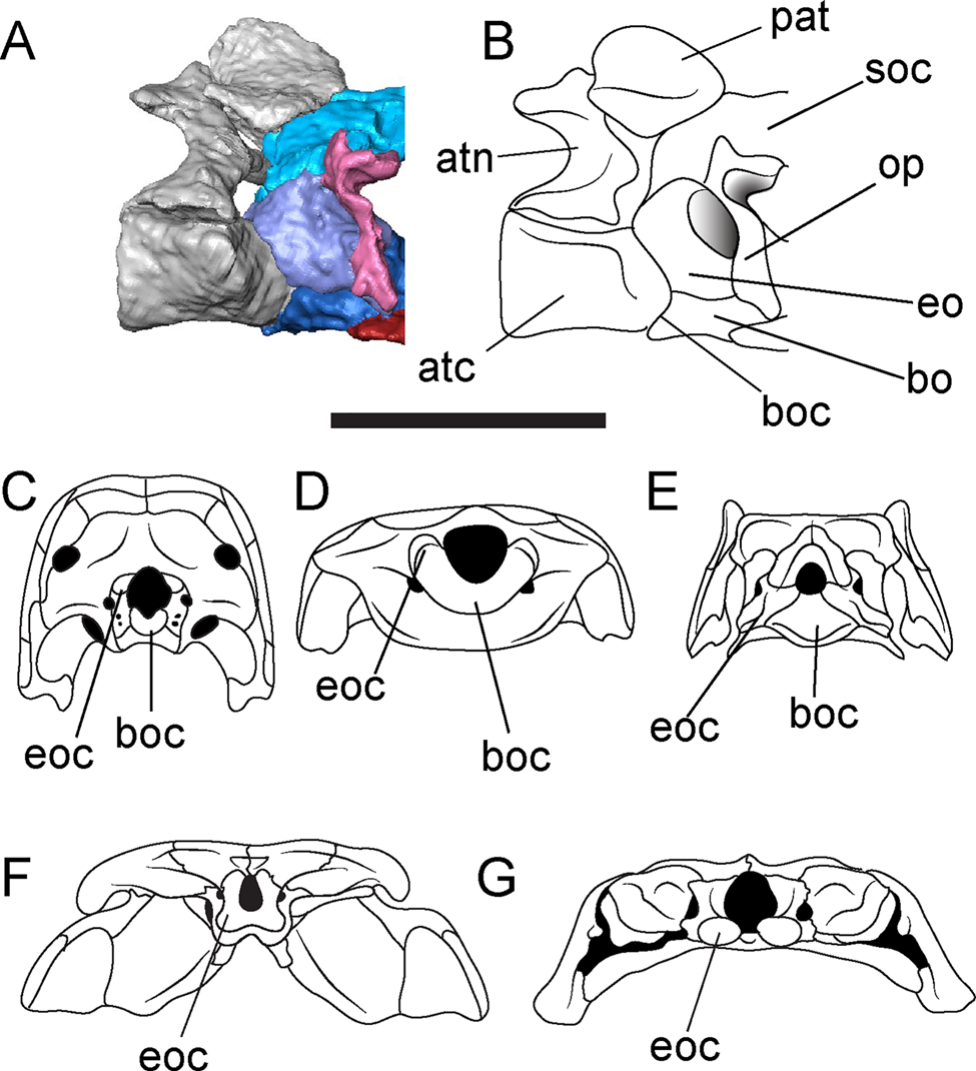
Atlantoccipital articulation of *Brachydectes newberryi*, and comparison with selected early tetrapods. **A-B**, atlantoccipital articulation of *Brachydectes newberryi*, KUVP 49541, in right lateral view; **A**, rendered from μCT; **B**, interpretive drawing; **C**, *Araeoscelis gracilis*, after [81], occipital view; **D**, *Quasicaecilia texana*, after [43], occipital view; **E**, *Brachydectes newberryi*, occipital view; **F**, *Acheloma dunni*, after [79], occipital view; **G**, *Hynobius amjiensis*, occipital view. **A-B**, scale bar equals 5 mm; **C-G**, not to scale. Abbreviations: **atc**, centrum of atlas; **atn**, neural arch of alas; **bo**, basioccipital; **boc**, basioccipital condyle or cotyle; **eo**, exoccipital; **eoc**, exoccipital condyle or shelf; **op**, opisthotic; **pat**, proatlas; **soc**, supraoccipital.

The atlantoccipital articulation in *Brachydectes* has been identified as exhibiting intermediate morphology between the atlantoccipital articulation of early tetrapods and the atlantoccipital articulation of modern lissamphibians [23, 25]. However, again, the detailed morphology presented here suggests strongly that this is not the case. The atlantoccipital articulation of lissamphibians consists of paired occipital condyles that form the primary articulation between the occiput and atlas. There is no basioccipital articulation with the atlas, nor is there a proatlantal arch at any stage of development. In *Brachydectes*, in contrast, the basioccipital cotyle forms the primary articulation with the atlas, with shelflike processes of the exoccipital forming accessory articulations with the atlas as well as the proatlantal arch (Fig 15). Small processes of the exoccipital are found in some early amniotes [81] and serve as a site of articulation with the proatlas. Additionally, although the basal amniote atlantoccipital articulation consists of a well-developed basioccipital condyle, a pit receiving the notochord is present within the basioccipital condyle in some modern reptiles (lost in birds and lepidosaurs) as well as many basal amniotes [82]. Although the presence of a proatlas itself is plesiomorphic for tetrapods, the presence of distinct processes of the occiput that articulate with it is not found within the amniote stem (e.g. seymouriamorphs and diadectomorphs) and in no taxon with exoccipital condyles do they articulate with the proatlas in any manner.

The evolution of the hypaxial cervical musculature in early tetrapods has been generally overlooked, although it has been discussed in some detail by Olson [83]. The hypaxial musculature of lissamphibians inserts along the ventral surface of the otic capsules, a feature shared with many early tetrapods [83]. In contrast, the cervical musculature of amniotes inserts on the basioccipital [29]. An occipital insertion of the hypaxial musculature has been recently identified in the microsaur *Quasicaecilia texana* [43], and paired fossae lateral to the basioccipital cotyle of *Brachydectes* suggest a similar amniote-like condition of the lysorophian occiput.

Although branching patterns of the internal carotids are highly variable in modern tetrapod taxa [84], the course of the common internal carotid and the location of the first cerebral branch of the internal carotid is generally conserved among early tetrapods [75, 85]. In lissamphibians, the common internal carotid enters the braincase through the basal plate of the parasphenoid or through the optic wall and then branches within the cavum cranii [86–87]. Branching of the common internal carotid into cerebral and palatal arteries outside of the skull, as in *Brachydectes*, is an apomorphy of the amniote stem and crown [75].

The presence of a well-developed crista interfenestralis in *Brachydectes* divides the otic capsule from the metotic fossa. Similar morphology of the otic region has recently been described in the microsaur *Dvellecanus carrolli* [42], as well as various amniotes [72, 75]. Importantly, the crista interfenestralis precludes a posterior course of the perilymph circulation, seen in all lissamphibians [1] and some temnospondyls [88], and thus suggests an anterior path of the perilymph circulation, as in amniotes.

The morphology of the anterior braincase, particularly the trabecular cartilages, has been a subject of some debate in the past [60] but has not been implemented in phylogenetic analyses to date. Lissamphibians, in contrast to amniotes, have broadly-spaced ethmoid trabeculae which never meet in the midline, forming a broad, flat, fenestrate base to the anterior braincase, a condition called platytraby [60]. In amniotes, the ethmoid trabeculae are fused posterior to the nasal capsules to form a trabecula communis for part or all of their length (tropitraby), producing a narrow, keeled floor of the braincase, often well-developed into an interorbital septum. Although at first glance, the broad, flat cultriform process of the parasphenoid of *Brachydectes* appears platytrabic, the presence of a well-developed midline presphenoid element suggests the presence of a trabecula communis, which could indicate that *Brachydectes* is tropitrabic. Similar morphology has been observed in a number of recumbirostrans as well [41–43]. It is unclear whether the basal condition in tetrapods is tropitraby or platytraby [61], but we consider it important to note that, while the broad cultriform process of the parasphenoid of *Brachydectes* is superficially similar to that of salamanders and caecilians, this does not reflect a lissamphibian-like organization of the neurocranium itself.

In summary, neurocranial morphology does not support a close relationship between *Brachydectes* and lissamphibians. The braincase of *Brachydectes* shows a number of synapomorphies associated with crown amniotes as well as recumbirostran ‘microsaurs,’ and lacks a number of neurocranial synapomorphies shared between lissamphibians and some temnospondyls. This may reflect broader phylogenetically-informative patterns in uncharacterized neurocranial variation in early tetrapods, in which case the fact that *Brachydectes* exhibits a braincase that is less amphibian-like and more amniote-like than the basal tetrapod condition will have to be addressed if the lepospondyl hypothesis of lissamphibian origins, as currently understood, is to remain viable.

#### *Brachydectes* and other lepospondyls

Morphology of the braincase of *Brachydectes* suggests a close relationship with the brachystelechid ‘microsaurs’ *Carrolla craddocki* [41] and *Quasicaecilia texana* [43], within the Recumbirostra. *Brachydectes* shares with *Carrolla* and *Quasicaecilia* a robust ossification within the columella ethmoidalis, a pillar-like pila antotica that braces against the skull roof, no participation of a descending flange of the frontal in the suture between the orbitosphenoid and the frontal, robust orbitosphenoid walls without fossae to accommodate the cerebral hemispheres or olfactory tracts, an anterior-facing fenestra antotica, passage of the trigeminal nerve through a foramen restricted to the ventral portion of the fenestra antotica, and relatively ventral position of the optic foramen. Additional cranial characteristics supporting this relationship include a reduced number of enlarged teeth in the maxilla and dentary, and a mandibular fenestra between the dentary, surangular, and angular. A close relationship between *Brachydectes* and brachystelechids has been suggested in the past [15–17] on the basis of similar reduction of the posterior skull and palate, as well as similarities in the axial and appendicular skeleton.

In contrast, the braincase of *Brachydectes* shows numerous dissimilarities to the braincases of aïstopods, with which it has been allied in the past [3, 21, 31, 33]. Anatomy of the braincase has been described in the aïstopods *Phlegethontia* [40] and *Sillerpeton permianum* [89]. In aïstopods, the antotic fissure faces laterally, and the foramen serving the trigeminal nerve is laterally directed as well. Furthermore, in aïstopods, the olfactory nerve exits the braincase far ventrally [40], whereas in *Brachydectes* and in microsaurs, the foramina serving the olfactory nerve are floored by the median anterior braincase bone and the cultriform process of the parasphenoid [33, 41–43]. In *Brachydectes* and in microsaurs, the basal plate of the parasphenoid covers most of the ventral surface of the otic region and participates in the ventral margin of the fenestra vestibuli [32, 33, 42], whereas in aïstopods, the parasphenoid is restricted to the sphenoid region only, leaving the otic capsules exposed ventrally [40].

A close relationship has also been found between *Brachydectes* and the Nectridea [3, 21, 31, 33], but is not supported by the morphology described here. The braincase is poorly known in most nectrideans, but has been partially described in diplocaulids [58, 90, 91]. *Brachydectes* differs from nectrideans in having ossified orbitosphenoid, basioccipital, and supraoccipital elements (all present in other microsaurs but absent in nectrideans). Nectrideans also demonstrate participation of the exoccipital in the fenestra vestibuli and full enclosure of the metotic foramen by the exoccipital (possibly associated with invasion of the ventral otic capsule by the exoccipital) [58, 90, 91], conditions not seen in *Brachydectes* nor any other recumbirostran.

Although no phylogenetic analysis is presented here, the overwhelming similarity between *Brachydectes* and brachystelechid microsaurs indicates a number of characteristics which will be incorporated into a future analysis currently in preparation. Moreover, the overwhelming dissimilarity between *Brachydectes* and both aïstopods and nectrideans suggests that prior skepticism towards a lysorophian-aïstopod clade (as seen in some phylogenetic analyses, such as [21]) may be well-founded, and that less-common phylogenetic results that unite lysorophians and brachystelechids (e.g. [15]) may in fact be correct. This would suggest that axial elongation was as common a trend among early tetrapods as among modern ones, possibly reflecting widespread ecological specialization.

### Is there evidence of miniaturization or neoteny in *Brachydectes?*

Reconstruction of a partial growth sequence is made possible by the substantial range of sizes in the sample of specimens examined here, with the smallest skull studied exhibiting a total skull length of 10.5 mm and the largest skull representing an animal with a skull length over 30 mm in length. Most skulls are approximately 10 mm or 20 mm in length; intervening sizes are not widely represented in this sample.

Ossification of all dermal bones has already been completed by the smallest studied specimen. In another lepospondyl (the aïstopod *Phlegethontia*) [40], a number of dermal bones ossify relatively late in development. Either this is not the case in *Brachydectes*, or else all specimens sampled here represent a relatively advanced stage of development, despite small size.

Ossification of some endochondral elements progresses through the ontogenetic sequence sampled here. In smaller specimens (e.g. DMNH 52081) the columella ethmoidalis is completely unossified. Ossification of this cartilage is partial in larger specimens (e.g. KUVP 49541), where it forms the medial surface of the foramen housing the olfactory nerve. Completion of ossification of the columella ethmoidalis is seen in the largest specimen (UNSM 32149), in which the entire space between the olfactory foramina is completely invaded by the presphenoid.

Complete ossification of the otic capsule is also ontogenetically delayed. The prootic and opisthotic are small floating elements in the smallest specimens, but ossify more completely at larger sizes, suturing to the parasphenoid, squamosal, and parietal in KUVP 49541, with many of the sutures completely obliterated in the largest specimen, UNSM 32149. In UNSM 32149, there is also a distinct crista parotica extending from the lateral wall of the prootic and opisthotic to brace against the squamosal; this structure is only weakly developed in KUVP 49541 and completely absent from smaller specimens.

Two specific structures initiate ossification late in ontogeny, and are observed only in UNSM 32149. The epipterygoid is fully ossified in UNSM 32149, but is unossified in all other specimens of *Brachydectes* studied here. Similarly, the distal portion of the columella, including its dorsal process, is only seen in UNSM 32149. In smaller specimens, the columella is a short, weakly-developed process extending from the footplate, and in the smallest specimens, the columella is essentially absent.

These observations present several implications. The first is that all but one of the *Brachydectes* specimens surveyed here likely represent immature specimens, with smaller specimens likely representing extremely immature animals. It is important to note that the majority of specimens of *Brachydectes* studied by previous authors [25–26, 28] are much smaller than UNSM 32149, and in some cases even smaller than the smallest specimens studied here. For characteristics that develop relatively late in ontogeny (the dorsal process of the columella, epipterygoid, crista parotica), descriptions focusing on juvenile material (and phylogenetic analyses relying on those descriptions) may miss important anatomical structures that were present in adult *Brachydectes*.

**Fig 16:**
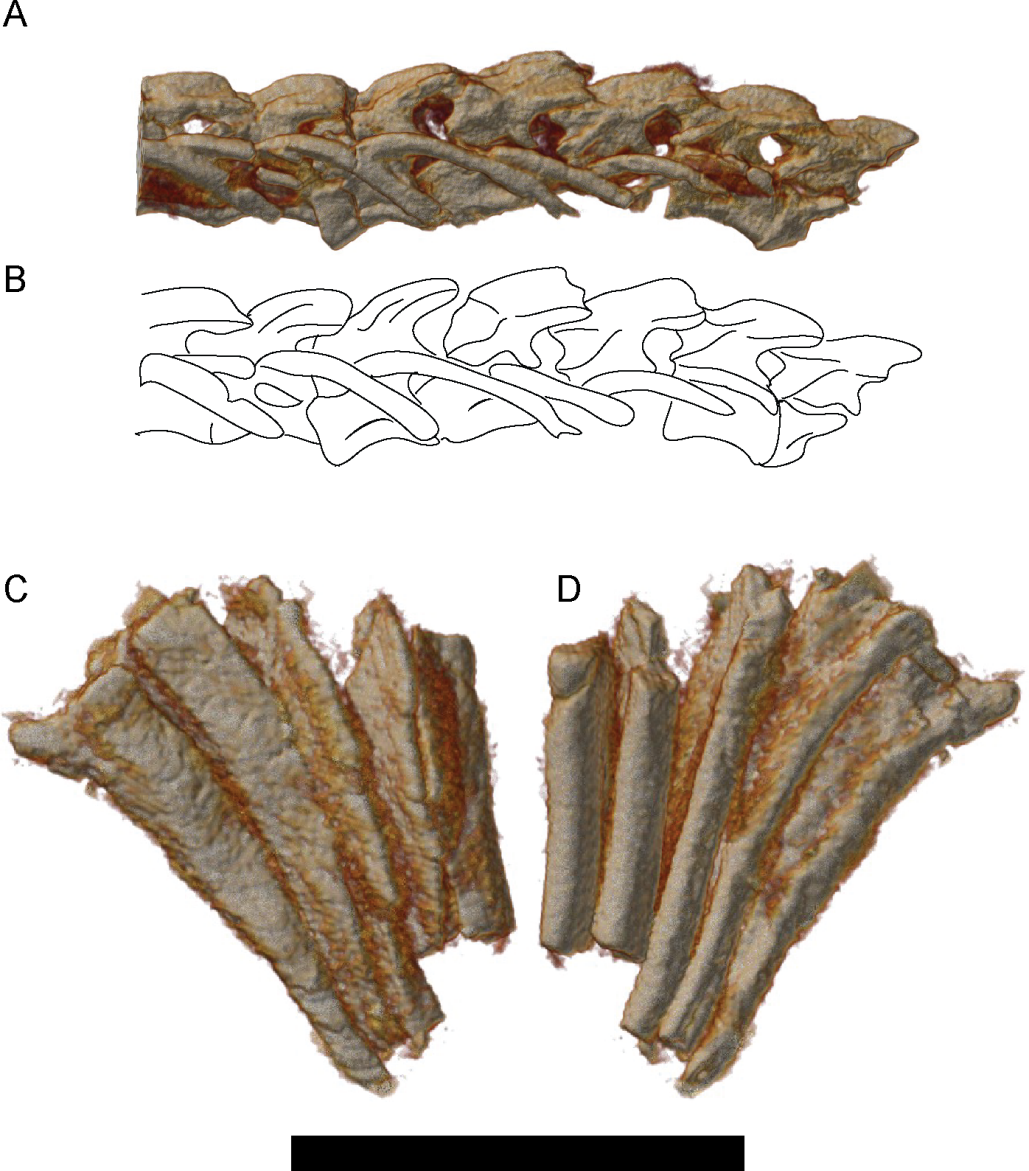
Rendered μCT volumes of selected elements of the axial skeleton of *Brachydectes newberryi*. **A-B**, dorsal vertebrae and ribs of a small specimen of *Brachydectes newberryi*, DMNH 51121, left lateral view; **A**, volume render; **B**, interpretive drawing; **C**, external and **D**, internal, view of four dorsal ribs of the largest specimen of *Brachydectes newberryi*, UNSM 32149. Abbreviations: **al**, rib alae; **na**, neural arch; **pl**, pleurocentra; **r**, rib. Scale bar equals 1 cm.

A second implication is that some of the taxonomic diversity previously identified among lysorophians may instead represent ontogenetic variation. Wellstead [28] reviewed lysorophian diversity and concluded that only three taxa could be consistently identified: *Brachydectes newberryi*, which primarily included small specimens with a skull length of less than one centimeter, *Brachydectes elongatus* (= *Lysorophus tricarinatus*), which primarily included specimens with a skull length between one and two centimeters, and *Pleuroptyx clavatus*, which consisted of a few very large specimens with a skull length likely exceeding two centimeters. Wellstead [28] diagnosed *Brachydectes* as distinct from *Pleuroptyx* on the basis of a broader pair of parietals, a more robust pectoral girdle, and well-developed alae on the ribs in the latter taxon. Parietal pair width appears to be variable, even within Wellstead’s sample, and does not reliably correspond with these other characteristics. Rib alae are observed in UNSM 32149 (Fig 16C and 16D) but not smaller specimens (Fig 16A and 16B), and may be ontogenetic as well. As such, *Pleuroptyx clavatus* appears to represent the large adult morphology of *Brachydectes newberryi* and may ultimately represent a junior synonym.

Within *Brachydectes*, Wellstead [28] identified two species: *B*. *newberryi* and *B*. *elongatus*. *B*. *newberryi* is distinguished from *B*. *elongatus* on the basis of the size of the lateral mandibular fenestra, the relative width of the parietal pair, the number of presacral vertebrae, and the presence of an unforked second ceratobranchial (“epibranchial” of [28]) in the latter taxon. The lateral mandibular fenestra does appear to decrease in size during ontogeny (Fig 12), and relative width of the parietal pair is highly variable, with substantial overlap within the material surveyed by Wellstead [28] as well as the material studied here. Variation in presacral vertebral counts reported by Wellstead [28] is likely indicative of interspecific variation, but it is difficult to align this with variation in cranial morphology. Few skulls described by Wellstead [28] and no skulls studied here are articulated with a complete set of presacral vertebrae, limiting the use of vertebral counts in species identification. The morphology of the second ceratobranchial does appear to differ between *B*. *newberryi* and *B*. *elongatus*, but it is unclear whether this represents taphonomic, ontogenetic, or intraspecific variation. If *Pleuroptyx clavatus* and *Brachydectes elongatus* are to be retained as valid taxa, rather than relegated as junior synonyms of *Brachydectes newberryi*, then these taxa will need to be revised and consistent morphological differences between them will have to be identified.

The limited ontogenetic timing data provides for some comparison with other early tetrapods. Anderson [40] has suggested that miniaturization in *Phlegethontia* may have been accomplished via early ossification of endochondral elements, which would have restricted later ossification of the dermal skeleton. The ossification sequence data reported here suggests that ossification in *Brachydectes* followed a different trajectory. Ossification of all dermal bones occurs very early in development, whereas ossification of some endochondral elements (specifically the presphenoid, otic capsule, stapedial columella, and epipterygoid) is delayed until late in skeletal maturity. Moreover, adult specimens of *Brachydectes* are large compared to many recumbirostrans, and are substantially larger than typical “miniaturized” taxa. We consider it unlikely that the relatively unique morphology exhibited by *Brachydectes* is the result of miniaturization alone.

The possibility that the unique morphology of *Brachydectes* reflects cessation of ontogeny at an early stage of development [6, 16–17] is also not supported by the morphology we present here. Several bones found in many early tetrapods are missing from the dermal skull of *Brachydectes* (the postfrontal, postorbital, jugal, and quadratojugal), and the maxilla is dramatically shortened, resulting in an open postorbital and temporal region that resembles that of neotenic salamanders. However, even small specimens of *Brachydectes* preserve the clavicle and scapula, elements which are considered indicative of skeletal maturity in other lepospondyls [10]. Additionally, *Brachydectes* demonstrates extensive ossification of endochondral bone (both in the chondrocranium and postcranium) in larger individuals, in contrast to the condition observed in neotenic salamanders. We thus consider it unlikely that the gross morphology of the skull of *Brachydectes* indicates a heterochronic origin of lysorophian skull morphology.

### Fossorial adaptations of *Brachydectes*

Previous workers [26, 28] have commented on possible fossorial adaptations of *Brachydectes*. The presence of distinct recumbirostran characteristics within the braincase permits a more precise discussion of how and why the morphology of *Brachydectes* differs from closely-related early tetrapods, and thus a better understanding of its ecology.

That *Brachydectes* inhabited burrows at least part of the year is supported by direct evidence; skeletons of *Brachydectes* are readily found within burrow structures [44–46, 92]. These burrows have generally been interpreted as estivation burrows [44–46] excavated in soft subaqueous sediment, partly due to the sedimentology of these localities [44–46] and partly due to the common interpretation of *Brachydectes* as an aquatic gill-breathing early tetrapod.

However, the skeletal evidence for fossoriality in *Brachydectes* has remained somewhat equivocal. Bolt and Wassersug [26] argued for a greater role of fossoriality in the ecology of *Brachydectes*, based on comparisons with modern amphisbaenian squamates, and specifying two general lines of anatomical evidence: the skull and palate were heavily reinforced to withstand compression and torsion stresses, and the jaw and suspensorium were modified to allow the mouth to open within enclosed spaces. Morphology identified in support of the former includes a dramatically thickened skull roof, deep sinuous sutures between skull roofing elements, and robust connections between the braincase, skull roof, and palate, whereas the latter is supported by the ventrally-recessed articular fossa and anteriorly-canted suspensorium. Bolt and Wassersug [26] also make note of the roughly wedge-shaped skull of *Brachydectes*, and a close relationship between the stapes and jaw articulation. They subsequently concluded that *Brachydectes* likely burrowed within soft sediment in freshwater environments [26].

Wellstead [28] rejected much of this argument, and argued that the morphology identified by Bolt & Wassersug [26] could be better explained by buccopharyngeal pumping (incorrectly identified by Wellstead as aquatic inertial feeding) rather than facultative or obligate fossoriality. Foremost among these characteristics is the anteriorly-canted suspensorium and ventrally recessed articular fossa, which Wellstead regarded as adaptations for increasing speed of jaw depression and gape size, respectively [28]. Structural adaptations involved in the reinforcement of the braincase were reinterpreted by Wellstead as adaptations associated with small size rather than fossoriality.

If, as suggested here, *Brachydectes* is ultimately found within the Recumbirostra, this permits more specific and constrained comparison of lysorophian morphology with the skulls of more conservative relatives, primarily the brachystelechids *Carrolla* and *Quasicaecilia*, for which skulls have recently been described in detail [41, 43], as well as the more generalized recumbirostrans *Nannaroter mckinzei* [71], *Huskerpeton englehorni* [33], *Rhynchonkos stovalli* [42], *Aletrimyti gaskillae* [42], and *Dvellecanus carrolli* [42].

Recent redescriptions of recumbirostran morphology have found strong morphological support for a fossorial lifestyle throughout this group [33, 41–43, 71]. In addition to gross morphology (shovel-like snout, expanded surfaces for insertion of epaxial musculature, shortened limbs, elongate body), a number of specific characteristics have been recognized. The anterior sphenoid and posterior ethmoid region are heavily ossified into presphenoid and mesethmoid bones, bracing the nasal region against dorsoventral compressive forces in several recumbirostrans [41–43]. The orbitosphenoids are also heavily ossified, and form an overlapping articulation with the skull roof (frontals and sometimes parietals) and enlarged cultriform process of the parasphenoid, in a ‘cranial box’ arrangement reminiscent of modern fossorial squamates [33, 41–43, 71]. The endochondral bones of the otoccipital region are also co-ossified in some forms [42–43], even incorporating the basisphenoid and parasphenoid in some taxa [41, 43]. Anterior displacement of the lower jaw permits jaw opening within the confines of a burrow [42], and is accomplished despite inferior mechanical advantage by an enlarged *m*. *depressor mandibulae* as indicated by the greatly enlarged retroarticular process seen in many recumbirostrans [42]. Similarly, jaw closure under inferior mechanical advantage would have been accomplished by expanded internal and external *mm*. *adductores mandibulae*, which would have been accommodated by the enlarged temporal emarginations seen in some recumbirostran taxa [33, 42, 70–71]. Conspicuous interdigitation and scarf joints within the skull roof would have served to resist deformation of the skull roof under compressive or torsional stress [41–42, 71].

Although the morphology of *Brachydectes* is consistent with these more general patterns, it differs from other recumbirostrans in a few key regards. In dorsal aspect, the skull is much longer and narrower than any other recumbirostran, especially in the temporal/otic region. This is accomplished by completely adpressing the squamosal and quadrate against the lateral wall of the otic capsule. The cheek is completely open from the orbit to the suspensorium, accommodating a greatly-enlarged *m*. *adductor mandibulae externus* (*mame*). An emarginated cheek is observed in various microsaurs, including hapsidopareiontids [32, 68–69], ostodolepids [32, 68, 71], *Tambaroter carrolli* [70], and *Huskerpeton englehorni* [33], but the condition seen in *Brachydectes newberryi* is exaggerated in comparison with other microsaurs, and extends dorsally to the parietals. This is partially accomplished via the loss of the quadratojugal, jugal, postorbital, and postfrontal, and via the modification of the squamosal into a thin strap restricted to the lateral surface of the quadrate. The cultriform process of *Brachydectes* is also laterally expanded in comparison with other microsaurs, including brachystelechids, making the orbitosphenoids and pleurosphenoids vertical rather than angled dorsolaterally. The bones of the skull roof (particularly the frontals, parietals, and postparietals) are thickened and strongly interdigitated, and articulate tightly with much of the dorsal portion of the braincase, suggesting further refinement of the ‘cranial box’ arrangement of more generalized recumbirostrans.

Morphology of the braincase in *Brachydectes* suggests greater resistance to dorsoventral forces on the skull centered on the frontals and parietals and a reduction of the role of a rostral scoop in excavation, suggesting a different mode of burrowing from shovel-snouted recumbirostrans such as *Pelodosotis* [32] or *Nannaroter* [71] as well as the four-step excavation cycle recently proposed for *Quasicaecilia texana* [43]. Skull morphology in modern fossorial squamates corresponds closely with burrowing mode [93, 94] and substrate type [95], permitting inference of burrowing mode and substrate type in extinct fossorial taxa such as *Brachydectes*. In gymnophthalmids and amphisbaenids, a steeply-inclined shovel-like skull is typically associated with burrowing in sandy substrate, whereas a lower, bullet-shaped skull is associated with burrowing in denser soil [95]. Bracing of the skull for dorsoventral compression is associated specifically with shovel-type burrowing modes [93, 96]. Axial elongation is also typical in amphibaenians employing shovel-type burrowing, permitting a greater total mass of the epaxial musculature associated with head lifting without also increasing the cross-sectional area of the body [97]. We consider the morphology of *Brachydectes* to be consistent with shovel-type headfirst burrowing in consolidated sediment.

This both clarifies and complicates interpretations of *Brachydectes* burrowing traces. Burrows containing lysorophian skeletons have been known for some time [27, 44–46, 92, 98]), and have been regularly cited as evidence of aestivation in this taxon. These burrows preserve irregularly-spaced nodes consistent with headfirst burrowing (specifically dorsad adpression), corroborating the osteology-based inference of burrowing mode. Although the burrows are traditionally interpreted as simple subaqueous excavations [45–45, 92], several features of these burrows are more consistent with excavations above the water table. Distinct node structures in the walls of lysorophian burrows are consistent with their excavation in consolidated sediment [45–46] and the presence of multiple individuals in some burrows [92] may support alternate interpretations of these burrow structures. Lysorophian burrows are also sedimentologically distinct from lungfish burrows, which preserve a mudstone-and-organic ‘shell’ surrounding the estivation chamber [45] and are found primarily on the edge of fossil ponds rather than within the center of them [46]. We do not argue here for a specific reinterpretation of lysorophian burrow traces, but rather caution against strong inference of lysorophian ecology and physiology based on prior interpretations of lysorophian burrowing behaviors.

## Supporting Information

S1 Table μCT scan parameters for *Brachydectes newberryi* specimens used in this study. **Abbreviations: DMNH**, Denver Museum of Nature and Science (Denver, Colorado, USA); **KUVP**, University of Kansas Natural History Museum and Biodiversity Institute, Vertebrate Paleontology Division (Lawrence, Kansas, USA); **UNSM**, University of Nebraska State Museum (Lincoln, Nebraska, USA).
(XLSX)
